# Interspecific and Geographic Variation in the Diets of Sympatric Carnivores: Dingoes/Wild Dogs and Red Foxes in South-Eastern Australia

**DOI:** 10.1371/journal.pone.0120975

**Published:** 2015-03-19

**Authors:** Naomi E. Davis, David M. Forsyth, Barbara Triggs, Charlie Pascoe, Joe Benshemesh, Alan Robley, Jenny Lawrence, Euan G. Ritchie, Dale G. Nimmo, Lindy F. Lumsden

**Affiliations:** 1 School of BioSciences The University of Melbourne, Victoria, Australia; 2 Arthur Rylah Institute for Environmental Research, Department of Environment, Land, Water and Planning, Heidelberg, Victoria, Australia; 3 'Dead Finish', Genoa, Victoria, Australia; 4 Parks Victoria, Bright, Victoria, Australia; 5 La Trobe University, Melbourne, Victoria, Australia; 6 Parks Victoria, Heyfield, Victoria, Australia; 7 Centre for Integrative Ecology and School of Life and Environmental Sciences, Deakin University, Burwood, Victoria, Australia; University of Sydney, AUSTRALIA

## Abstract

Dingoes/wild dogs (*Canis dingo*/*familiaris*) and red foxes (*Vulpes vulpes*) are widespread carnivores in southern Australia and are controlled to reduce predation on domestic livestock and native fauna. We used the occurrence of food items in 5875 dingo/wild dog scats and 11,569 fox scats to evaluate interspecific and geographic differences in the diets of these species within nine regions of Victoria, south-eastern Australia. The nine regions encompass a wide variety of ecosystems. Diet overlap between dingoes/wild dogs and foxes varied among regions, from low to near complete overlap. The diet of foxes was broader than dingoes/wild dogs in all but three regions, with the former usually containing more insects, reptiles and plant material. By contrast, dingoes/wild dogs more regularly consumed larger mammals, supporting the hypothesis that niche partitioning occurs on the basis of mammalian prey size. The key mammalian food items for dingoes/wild dogs across all regions were black wallaby (*Wallabia bicolor*), brushtail possum species (*Trichosurus* spp.), common wombat (*Vombatus ursinus*), sambar deer (*Rusa unicolor*), cattle (*Bos taurus*) and European rabbit (*Oryctolagus cuniculus*). The key mammalian food items for foxes across all regions were European rabbit, sheep (*Ovis aries*) and house mouse (*Mus musculus*). Foxes consumed 6.1 times the number of individuals of threatened Critical Weight Range native mammal species than did dingoes/wild dogs. The occurrence of intraguild predation was asymmetrical; dingoes/wild dogs consumed greater biomass of the smaller fox. The substantial geographic variation in diet indicates that dingoes/wild dogs and foxes alter their diet in accordance with changing food availability. We provide checklists of taxa recorded in the diets of dingoes/wild dogs and foxes as a resource for managers and researchers wishing to understand the potential impacts of policy and management decisions on dingoes/wild dogs, foxes and the food resources they interact with.

## Introduction

Large- and medium-sized mammalian carnivores influence ecosystem structure and function worldwide through predation and intraguild competition [1, 2]. The impacts of introduced mammalian carnivores on native prey often exceed those of native carnivores, regularly causing their decline or extinction [3–7]. In addition, human-carnivore conflicts are increasing globally [1, 2] due to actual or perceived impacts of carnivores on livestock [8–10]. Managing the impacts of carnivores is therefore important in both agricultural and conservation settings.

Mammalian carnivore species often co-occur, potentially creating complex interspecific interactions and trophic dynamics. If sympatric carnivores use the same food resources, interactions can range from commensalism to mutualism to competition and predation [11–15]. The relative size of co-occurring carnivores contributes to these interactions. For instance, carnivore size determines the upper size limit of prey species and contributes to mortality rates across potential prey species [16]: the dietary niche of smaller carnivores is often nested within the range of prey consumed by larger species [17, 18], although a dietary shift in large carnivores towards hypercarnivory and specialisation on large prey can result in dietary separation from smaller carnivores that subsist on much smaller prey [19]. Interspecific killing within carnivore guilds, which contributes to different mortality rates across carnivore species, is also related to body size [20], and may be symmetrical (both species kill each other) or asymmetrical (one species kills the other) [21]. However, carrion can form an important component of the diet of carnivores, which may scavenge on the carcasses of animals much larger than themselves [22, 23].

Understanding the diets of carnivores is important for two main reasons. First, such knowledge can identify prey species that may be impacted by predation. Prey of particular interest are: (i) native species that may be negatively impacted by carnivores [3, 24–27], (ii) species that can become overabundant when carnivores are controlled (e.g., large macropods in Australia [28, 29–31]), and (iii) domestic livestock species that have economic and/or social value for people [1, 27, 32]. Second, a knowledge of carnivore diets can help managers to forecast the consequences of changes in food resource availability (e.g., due to culling of prey species or the establishment of new prey species [33]) and undertake integrated pest management [34].

As with many areas of the world, south-eastern Australia has a suite of native and introduced mammalian carnivores [35]. Of particular interest are dingoes (*Canis dingo*), ‘wild’ dogs (*C*. *familiaris*) and hybrids of the two (10–25 kg [36, 37]), and red foxes (*Vulpes vulpes* [5–8 kg]) because of their perceived and actual impacts on native mammals and domestic livestock [38, 39–42]. Dingoes have been present on mainland Australia for at least 4000 years [43], but wild dogs have established more recently following European contact [39]. Red foxes were introduced to Australia in the 1860s [44] and are now widespread in southern mainland Australia [38].

Dingoes, wild dogs and their hybrids (hereafter referred to as ‘wild dogs’ because their scats are indistinguishable) and foxes consume predominantly mammalian prey [45]. However, there is evidence of niche partitioning based on prey size [46, 47]. Although both wild dogs and foxes eat medium-sized mammalian prey [47], some studies suggest that wild dogs eat more large prey and less small prey than foxes [48–51]. Wild dogs may pose risks to native fauna through predation [26, 52], yet their presence may have indirect and net biodiversity benefits, particularly for small native fauna, through suppression of populations of red foxes and feral cats (*Felis catus*) [53, 54] (mesopredator release hypothesis [55]). Further, wild dogs may limit the abundance of large herbivores, both native (e.g., kangaroos (*Macropus* spp.) [31, 56]) and introduced (e.g., feral goats (*Capra hircus*) and feral pigs (*Sus scrofa*) [54]), which can negatively impact biodiversity when overabundant [57]. However, wild dogs also kill domestic sheep (*Ovis aries*), cattle (*Bos taurus*) and goats [39, 58–60], and because of this they are subject to lethal control in much of south-eastern Australia [39, 61].

Predation by red foxes has been implicated in the decline and extinction of numerous small native mammals, particularly within the Critical Weight Range (CWR: 35–5500g) [62–66]. Consequently, foxes are listed as a key threatening process in state and federal legislation [67, 68]. Foxes also kill lambs [8, 69]. Hence, foxes are widely controlled in agricultural [38, 70] and native habitats [71] in south-eastern Australia [72].

Given the potential impacts of wild dog and fox predation on native and introduced species, and the costs of controlling these impacts, it is important to understand the dietary niches of these two taxa. Here, we used scat contents to evaluate interspecific and geographic variation in the diets of wild dogs and foxes in Victoria, south-eastern Australia. We tested six predictions:

1. The diets of wild dogs and foxes overlap and diet breadth is greater for foxes.
2. The diets of wild dogs and foxes vary geographically.
3. The diet of wild dogs is composed of larger mammals than the diet of foxes and the size-range of mammals in the diet of foxes is nested within the diet of wild dogs.
4. The diet of wild dogs is composed of more domestic livestock (i.e., sheep and cattle), large introduced herbivores (e.g., sambar deer (*Rusa unicolor*)), and large native herbivores (kangaroo species) than the diet of foxes.
5. The diet of foxes contains more native CWR mammal species than the diet of wild dogs.
6. That intraguild predation between wild dogs and foxes is asymmetrical: the smaller fox occurs in the diet of the larger wild dog but not *vice versa*, and both wild dogs and foxes consume feral cats.

Finally, we provide checklists of taxa recorded in the diets of wild dogs and foxes in Victoria.

## Materials and Methods

### Study area

**Fig 1:**
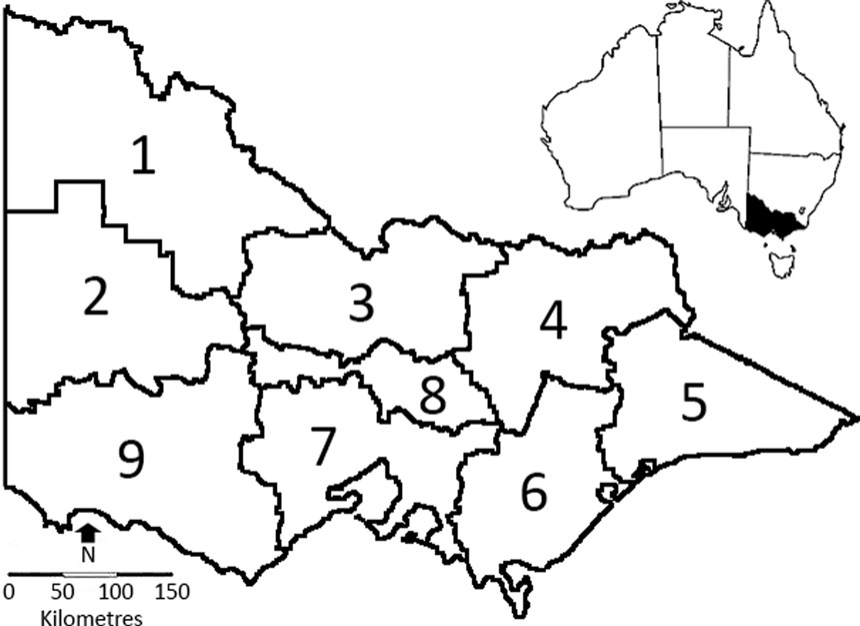
The State of Victoria, south-eastern Australia. The nine Victorian regions defined by the Australian Government Bureau of Meteorology are: 1, Mallee; 2, Wimmera; 3, Northern Country; 4, North East; 5, East Gippsland; 6, West and South Gippsland; 7, Central; 8, North Central; and 9, South West.

**Fig 2:**
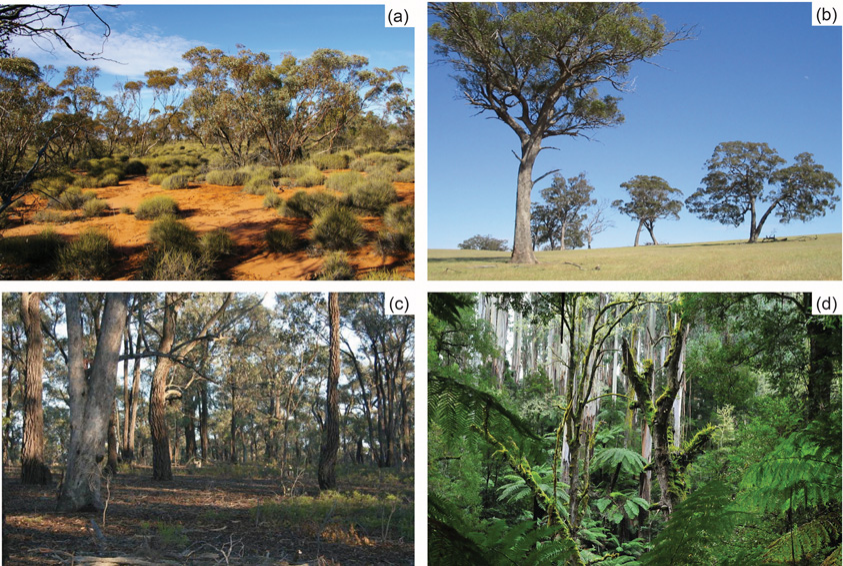
Photographs demonstrating ecosystem variation within the State of Victoria: (a) semi-arid Mallee ecosystem (Photo: Mallee Fire and Biodiversity Project Team); (b) farmland (Photo: Jim Radford); (c) Box Ironbark forest (Photo: Andrew Bennett); (d) wet forest (Photo: Wiki commons).

The State of Victoria (237,629 km^2^; Fig. 1) includes a wide variety of ecosystems, ranging from beaches (i.e., 0 m a.s.l.) and coastal forests to semi-arid rangelands to montane forests and alpine grasslands (1986 m a.s.l.) [73] (Fig. 2). We used nine regions defined by the Australian Government Bureau of Meteorology [74] (Fig. 1) to analyse spatial variation in the diets of wild dogs and foxes. These regions were selected for three main reasons. First, they approximately align with Victorian topographic, climatic, land-use and biological regions [75]. Second, their broad scale minimised problems associated with uncertainty regarding the exact location at which samples were collected and the relationship between the site at which samples were collected and locations at which food was consumed [49]. Third, they allow managers and researchers to easily find diet information relevant to their region(s) of interest.

### Data collation

We contacted managers, researchers and analysts who had collected and/or analysed the contents of scats collected from wild dogs and foxes in Victoria. We were provided with carnivore scat analysis data (5875 individual wild dog scats and 11,569 fox scats) collected from throughout Victoria during summer, autumn, winter and spring between 1983 and 2014 (Table 1). Analysts had identified food items in the diets of wild dogs and foxes using distinguishing macroscopic and microscopic features of indigestible remains in faecal matter, particularly morphometric features of mammalian hair, bones and teeth [76]. Eighty six percent of scat samples had been assigned as ‘definitely’ from a wild dog or from a fox, based on the presence of grooming hairs in scats and distinguishing scat characteristics, and 14% had been assigned as ‘probably’ from a wild dog or from a fox, based on the best match to scat characteristics. We excluded scats that had been collected from domestic dogs (i.e., scats from metropolitan areas and/or scats containing pet food). We also used four electronic databases (Web of Science, Ecology Abstracts, Google Scholar and Scopus), two library catalogues (The University of Melbourne and Victorian Government Library Service) and our personal networks to identify published and unpublished reports, journal articles and books containing information on wild dog and fox diet in Victoria from scat analyses. We included several studies that combined information from analyses of scat and stomach contents.

**Table 1:** Numbers of wild dog and fox scats collected in nine Victorian regions and all regions pooled (including additional samples that could not be allocated to a region).

Taxa	Sample type	Mallee	Wimmera	Northern Country	North East	East Gippsland	West/South Gippsland	Central	North Central	South West	Total (all regions)
Wild dog	scat	819	35	44	2668	1137	416	360	200	93	5875
Fox	scat	5592	189	98	2931	1064	463	629	181	308	11,569

The locations of scat samples were determined from site descriptions associated with diet analysis data, through cross-referencing with samples entered into the Victorian Biodiversity Atlas [77], and through discussions with staff responsible for sample collection or with knowledge of the areas where samples had been collected. Through this process it was determined that both wild dog and fox samples had been collected in each of the nine Victorian regions.

### Data analyses

Our metric for examining regional variation in the diets of wild dogs and foxes, and to compare the use of mammalian food items from different size-classes by wild dogs and foxes, was percentage frequency occurrence (hereafter ‘frequency occurrence’) of diet categories for each carnivore per region. The frequency occurrence, *F*, of a given food item *i* is defined as the number of samples *N* in which that food item occurs, expressed as a frequency of the total number of samples (or of the number of samples that contained food) [78]:

$$F_{i}=\left(\frac{N_{i}}{N}\right)\times 100.$$

The total frequency occurrence of dietary categories can exceed 100% due to the occurrence of multiple food items in some samples. Frequency occurrence is the most commonly used method of interpreting scat-analysis data, and is recommended when quantitative information on scat composition is unavailable [27]. It provides similar diet estimates to percentage volume measures for the more important food items [76], and it can be applied to all food types. Frequency occurrence can provide useful information on the range of foods eaten, including rare food items [27] and spatial and temporal variation in their frequency in the diet [48].

We defined eight diet categories: large mammal (≥7.0 kg), medium mammal (0.5–6.9 kg), small mammal (<0.5 kg), bird, reptile/amphibian, insect, other invertebrate and plant material. The mammal size classes follow Pascoe et al. [79] and are similar to those used by others [80, 81]. Frequency occurrence estimates were based on all samples because it was often not possible to distinguish between studies that considered only mammals/vertebrates and those that considered all diet categories.

Mammalian food items were placed in a size-class according to mean adult body masses obtained from Menkhorst and Knight [82]. Where mean masses were unavailable, the mid-point of the adult mass range provided by Menkhorst and Knight [82] was used. For species that Menkhorst and Knight [82] provided maximum mass limits only, additional information was used to establish a mean mass, or a mass range from which the mid-point could be taken as follows: black wallaby (*Wallabia bicolor*) [83], eastern grey kangaroo (*Macropus giganteus*) [84, 85], western grey kangaroo (*M*. *fuliginosus*) [86], horse (*Equus caballus*) [87], koala (*Phascolarctos cinereus*) [88], red kangaroo (*M*. *rufus*), eastern wallaroo (*M*. *robustus robustus*) and sheep [89]. While many mammal species can be accurately identified from hair samples, this is not always possible [90] and it was therefore necessary to lump some food items into genera or families. In these instances, food items were classified according to the size of the majority of species recorded in diets in a genus or family (e.g. ‘undetermined Dasyuridae’ were classified as ‘small’ because all Dasyuridae species identified in diets except spot-tailed quoll (*Dasyurus maculatus*) were classified as small). The exceptions were glider species that could not be distinguished between the genera *Petauroides* and *Petaurus*. These taxa were classed as ‘medium’ because the species identified in the diets from these genera were evenly distributed in the small- and medium-sized classes.

We identified mammalian food items that occurred at a frequency of >10% across regions in the diet of wild dogs, and those that occurred at a frequency of >10% across regions in the diet of foxes. For both wild dogs and foxes, we then compared the frequency (counts) of these food items among the nine Victorian regions using the chi-square test for homogeneity [91]. Brushtail possum species (*Trichosurus* spp.) were amongst the more frequent prey items for both wild dogs and foxes. Because hair of mountain brushtail possum (*Trichosurus caninus*) and common brushtail possum (*Trichosurus vulpecula*) are difficult to distinguish [90] and many raw data records were identified to genus level only, records identified to genus and to species level within this genus were pooled for consideration as key mammalian food items.

Frequency occurrence suffers from several biases, particularly when comparing mammal items of different size in the diet [92–94]. Linear regression models developed from feeding trials can be used to estimate the biomass of food items consumed, eliminating several of the biases associated with frequency occurrence and providing a better approximation of the diet of carnivores [27]. We therefore converted wild dog and fox scat data to relative biomass and number of individuals consumed for each mammalian species, with the exception of brushtail possum spp. which we again considered in combination, across Victoria and within each region (S1 Appendix). For the reasons detailed in S1 Appendix, we used these metrics to compare the use of food items of interest (livestock, large introduced herbivores, large native herbivores (macropods with mean body mass >25 kg), native species in the CWR, and medium and large carnivores) by wild dogs and foxes, and by each carnivore among regions. Although the CWR overlaps with our small and medium dietary categories, this category was considered separately for native species given the high susceptibility of species in this weight range to extinction [62–66]. We used the linear regression model developed by Floyd et al. [92] for the wolf (*Canis lupus*) with modifications by Weaver [93]:

Y=0.439+0.008X,

where *Y* is the mass of the mammalian food item per scat, and *X* is the mean mass of an individual of a given mammalian food item. We multiplied *Y* by the number of scats in which each mammalian food item was recorded to estimate the relative total mass of each mammalian food item consumed per region, and then divided this value by the body mass of individuals of each mammalian prey species to estimate the relative number of individuals of each species consumed per region, as represented by the total sample of scats [92]. To provide a standardised measure of mass and number of individuals per scat, we then divided estimates of relative total mass and number of individuals of each mammalian food item by the scat sample size for that region.

Despite the potential for error associated with differential digestion between carnivore families [95] we applied Floyd [92] and Weaver’s [93] model to both wild dogs and foxes. We chose this approach in preference to the use of diet correction factors determined for the red fox [96–98] for three reasons. First, we did not want to obscure interspecific differences by using biomass estimation approaches with different biases. Second, our focus was on the mammalian component of the diet. Third, extrapolation of correction factors to prey not used in feeding trials may introduce errors [27].

The three estimates of diet (i.e. frequency occurrence, relative total biomass, and relative number of individuals of each prey type consumed) were compared graphically for all mammalian prey items identified to species level in wild dog and fox diets across Victoria to explore the relationships between these metrics. We provide a description of this comparison, and explanation of our selection of the metric deemed most appropriate to examine each of our six predictions, in S1 Appendix. To evaluate the potential impacts of wild dogs and foxes on species of conservation significance, we first estimated the total number of individuals (standardised per scat) of native mammalian species in the CWR (i.e., the sum of individuals consumed across species, excluding *Rattus* spp. identified to genus only as these could not be designated as native or introduced) consumed. We then graphically compared the number of individuals consumed by wild dogs and foxes Victoria-wide for CWR mammalian species of conservation significance. We used relative biomass to evaluate regional and interspecific differences in key mammalian food items (S1 Appendix). Key mammalian food items were considered to be those that were estimated to constitute ≥0.04 kg prey mass per scat. This mass was chosen as a threshold because in many regions there appeared to be a natural break at this point between the few dominant food items in the diets of wild dogs and foxes, and the large number of items for which relative biomass was considerably lower. We estimated the mean number of individuals of small, medium and large native mammals in the diets of wild dogs and foxes per 1000 scats. We also graphically compared the mass (per scat) of eutherian carnivores in the diets of wild dogs and foxes among regions. The occurrence of wild dog hair in wild dog samples, and fox hair in fox samples, can be attributed to cannibalism or grooming based on the quantity of hairs present: the presence of only a few (<10) hairs is indicative of ingestion of hairs while grooming, whereas the presence of a large number of hairs suggests the occurrence of predation or scavenging. However, the frequency of cannibalism could not be estimated because data sources did not always distinguish between records of grooming hairs and food items.

We estimated diet niche overlap between wild dogs and foxes and niche breadth for each species across Victoria and within each region, based on use of the eight diet categories (food resources): large mammal, medium mammal, small mammal, bird, reptile/amphibian, insect, other invertebrate and plant material. To estimate diet overlap we used Pianka’s index [99]:

$${\hat{O}}_{jk}=\frac{\sum_{i}^{n}{\hat{p}}_{ij}{\hat{p}}_{ik}}{\sqrt{\sum_{i}^{n}{\hat{p}}_{ij}^{2}\sum_{i}^{n}{\hat{p}}_{ik}^{2}}}$$

where *O*_*jk*_ = Pianka's measure of niche overlap between species *j* and species *k*; *p*_*ij*_ = proportion resource *i* is of the total resources used by species *j*; *p*_*ik*_ = proportion resource *i* is of the total resources used by species *k*; *n* = total number of resources. The value of this index ranges from 0 (no overlap) to 1 (complete overlap). Niche breadth (*B*_*A*_) was calculated using Levins’ standardized measure to compare the proportion (based on frequency occurrence) of food resources exploited by each carnivore [100, 101]:

$$B_{A}=\frac{\left(1/\sum p_{i}^{2}\right)-1}{n-1}$$

where *p*_*i*_ = proportion of occurrence of each food resource in the diet and *n* = the number of possible resources. This measure of niche breadth ranges from 0 to 1, with a value close to 0 indicating a narrow niche and a value close to 1 indicating a broad niche. The *B*_*A*_ index gives more weight to commonly eaten resources and less weight to rarely consumed resources [101]. To determine whether the mammalian component of the diet of foxes was nested within the mammalian component of the diet of wild dogs we compared the range of body masses of prey consumed by each carnivore [18].

### Checklists of species recorded in the diets of wild dogs and foxes in Victoria

We compiled our data into checklists of taxa recorded in the scats of wild dogs and foxes in Victoria. We summarised scat data as frequency occurrence for each item recorded as ‘definite’ or ‘probable’ prey in the diets of wild dogs and foxes within each of the nine regions and in all regions combined. Summary data from the literature, which largely consisted of unpublished reports, were used to compile presence records for the checklists but were not included in estimates of frequency occurrence of dietary items. In the checklists we identified native species of conservation significance that are listed under state and federal legislation [68, 77] and/or on the International Union for Conservation of Nature red list [102].

## Results

### Taxa recorded in the diets of wild dogs and foxes

Our checklists (S1 and S2 Tables) indicate that a minimum of 65 and 71 vertebrate taxa have been recorded in the diets of wild dogs and foxes, respectively, in Victoria. Fifty-seven mammal species (43 native and 14 introduced species) from 41 genera and 25 families (primarily Muridae, Macropodidae and Dasyuridae) were identified in the diet of wild dogs (S1 Table). Most (99%) bird remains in the diet of wild dogs were identified only to Class, however three bird species (three families) were recorded (S1 Table). Three reptile genera (three families) were recorded in the diet of wild dogs, and there were invertebrates from a minimum of seven families (S1 Table).

Sixty-two mammal species (48 native species and 14 introduced species) from 42 genera and 25 families (mostly the same families recorded for wild dogs) were identified in the diet of foxes (S2 Table). Five bird species (five families) were recorded in fox scats or stomachs in Victoria in the literature, but all bird remains recorded during this study were identified to Class only (S2 Table). Two reptile families were recorded in the diet of foxes (S2 Table). More insects (three species and eight families) were identified in the diet of foxes than in the diet of wild dogs (S2 Table). Excluding insects, invertebrates from a minimum of three other families were recorded in the diet of foxes, of which two crustaceans were identified to species (S2 Table).

**Fig 3:**
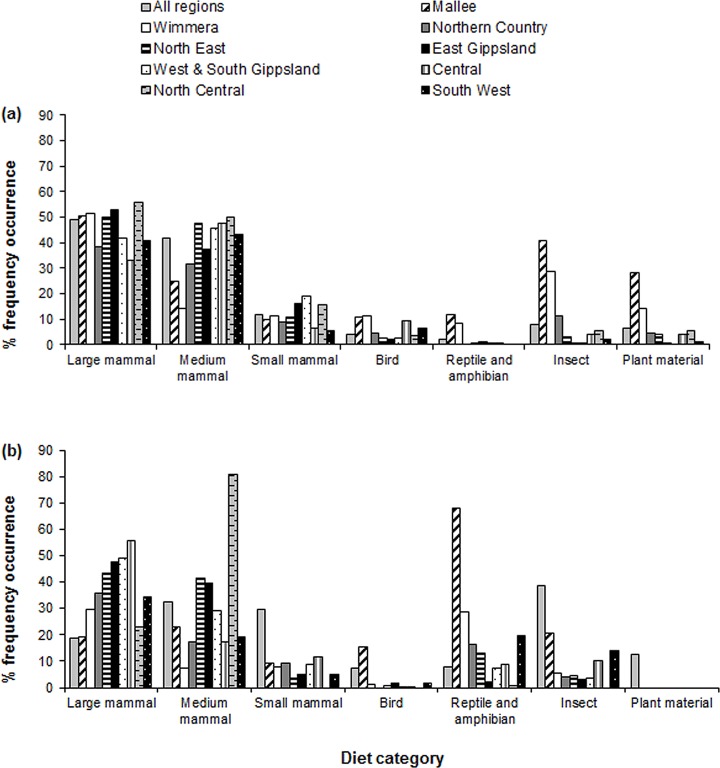
Percentage frequency occurrence of seven diet categories recorded in (a) wild dog scat, and (b) fox scat samples collected during 1983–2014 in nine Victorian regions and all regions pooled: Mallee; Wimmera; Northern Country; North East; East Gippsland; West and South Gippsland; Central; North Central; and South West. Sample sizes are provided in Table 1.

Mammals were the most frequently recorded items in the diets of wild dogs and foxes in Victoria (94% and 76%, respectively; S1 and S2 Tables; Fig. 3). Our checklists document the occurrence of several mammal species that to our knowledge have not previously been recorded in the published literature on the diets of these two carnivores in Victoria (S1 and S2 Tables). Across Victoria, other broad prey categories including birds, reptiles, amphibians and plant material, were recorded at low frequencies in the diet of wild dogs (<7%) but at moderate frequencies (7–13%) in the diet of foxes (S1 and S2 Tables; Fig. 3). Invertebrates other than insects were recorded at extremely low frequencies (<0.2%) in the diets of both species (S1 and S2 Tables). In contrast, insects were recorded in the diet of foxes (39%) in frequencies exceeding that of any single size-class of mammals, but were less frequently recorded in the diet of wild dogs (8%; Fig. 3).

### Diet overlap and niche breadth

There was high diet overlap (*O*_*jk*_ = 0.73) between wild dogs and foxes when data from all regions were pooled (Table 2). However, diet overlap varied among regions from a low of 0.40 in the North Central region to a high of 0.97 in the Northern Country region.

**Table 2:** Niche overlap (Pianka’s index; *O*_*jk*_) and breadth (Levins’ standardised measure; *B*_*A*_) of wild dog and fox diet in nine Victorian regions and all regions pooled, based on analysis of scats collected during 1983–2014.

Taxa	Niche parameter	All regions	Mallee	Wimmera	Northern Country	North East	East Gippsland	West/South Gippsland	Central	North Central	South West
Wild dog/Fox	Overlap: *O*_*jk*_	0.73	0.87	0.93	0.97	0.71	0.74	0.94	0.90	0.40	0.84
Wild dog	Breadth: *B*_*A*_	0.35	0.60	0.52	0.38	0.27	0.25	0.28	0.30	0.31	0.25
Fox	Breadth: *B*_*A*_	0.63	0.53	0.46	0.50	0.36	0.31	0.40	0.36	0.09	0.58

Niche overlap and breadth used data on frequency occurrence of eight diet categories: large mammal, medium mammal, small mammal, bird, reptile/amphibian, insect, other invertebrate and plant material). Sample sizes are provided in Table 1.

Niche breadth (*B*_*A*_) was greater for the fox than for the wild dog when data from all regions were pooled and in all regions except the Mallee, Wimmera and North Central regions (Table 2). Wild dog niche breadth was greatest in the Mallee and narrowest in the East Gippsland and South West regions (Table 2). Fox niche breadth was greatest in the South West region and narrowest in the North Central region (Table 2). Mammalian prey recorded in the diet of wild dogs ranged from a mean body mass of 0.008 kg to 500 kg, and in the diet of foxes ranged from 0.008 kg to 400 kg.

### Regional variation in the diets of wild dogs and foxes

**Fig 4:**
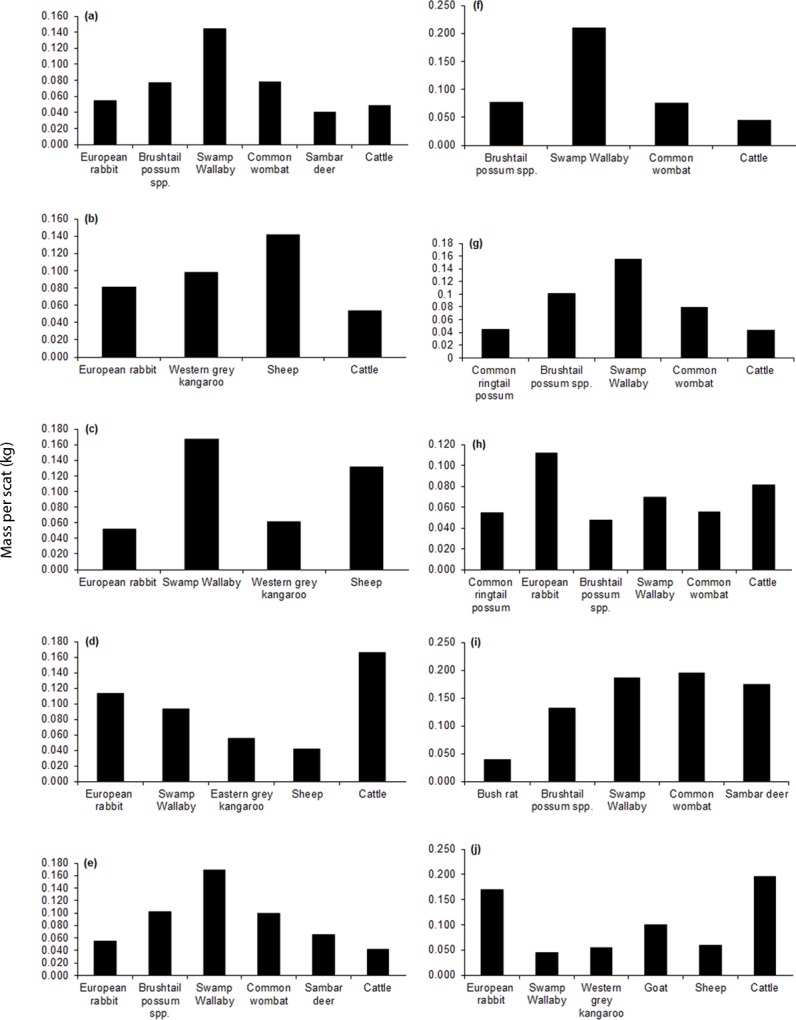
Regional variation in intake of key mammalian food items (≥0.04 kg prey biomass per scat) by wild dogs based on analysis of scats collected during 1983–2014 in (a) all Victorian regions, and in the (b) Mallee, (c) Wimmera, (d) Northern Country, (e) North East, (f) East Gippsland, (g) West and South Gippsland, (h) Central, (i) North Central, and (j) South West regions. Sample sizes are provided in Table 1.

**Fig 5:**
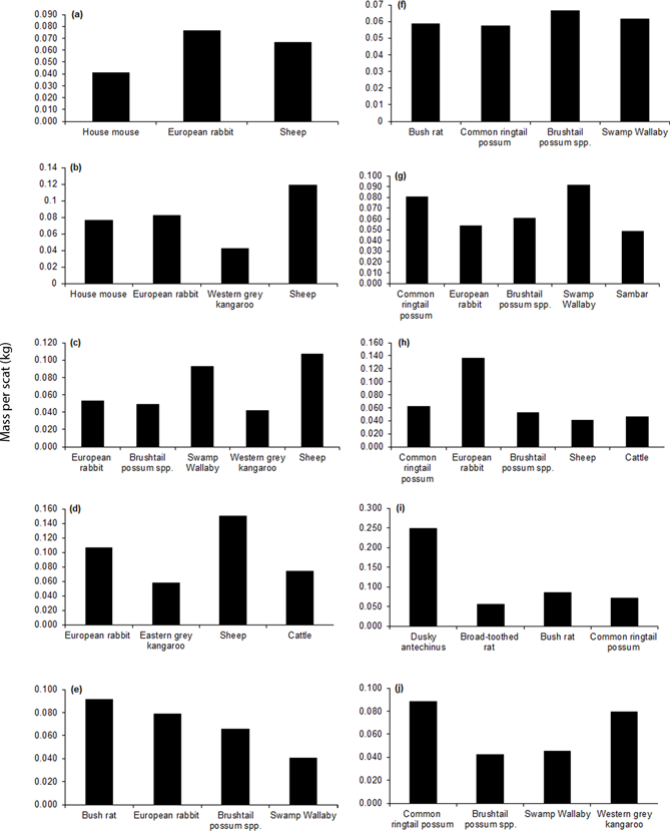
Regional variation in intake of key mammalian food items (≥0.04 kg prey biomass per scat) by foxes based on analysis of scats collected during 1983–2014 in (a) all Victorian regions and in the (b) Mallee, (c) Wimmera, (d) Northern Country, (e) North East, (f) East Gippsland, (g) West and South Gippsland, (h) Central, (i) North Central and (j) South West regions. Sample sizes are provided in Table 1.

**Fig 6:**
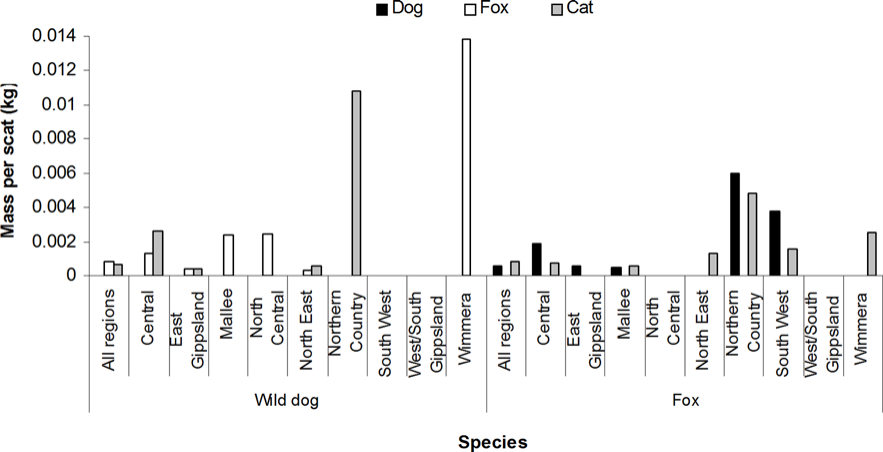
Mass (kg per scat) of eutherian predator species in the diets of wild dogs (*n* = 5148) and foxes (*n* = 11,143) in Victorian regions, based on analysis of scats collected during 1983–2014.

There was substantial regional variation in the diets of wild dogs and foxes (S1 and S2 Tables; Tables 2–4; Figs. 3–6). Across regions, the frequency of medium and large mammals in wild dog diet was high (32–56%), except in the Mallee and Wimmera regions where the frequencies of medium mammals were lower (25% and 14%, respectively; Fig. 3). The frequency of large mammals in the diet of wild dogs was lowest (33%) in the Central region (Fig. 3). The frequency of small mammals in the diet of wild dogs was generally intermediate (9–21%) across regions, but was much lower in the Central (6%) and South West regions (5%) (Fig. 3).

In the diet of foxes, the frequency of small mammals varied more than ten-fold across regions, from 7% in the Wimmera region to 81% in the North Central region (Fig. 3). The frequency of medium mammals in the diet of foxes was more consistent than small mammals, but still varied regionally; the greatest frequencies were in the East Gippsland (48%), West and South Gippsland (49%) and Central (56%) regions (Fig. 3), whereas the lowest frequencies were in the Mallee (19%) and North Central (23%) regions (Fig. 3). Large mammals were generally less frequent in the diet of foxes than small- or medium-sized mammals, ranging from moderately frequent in the Wimmera (39%) and Northern Country (32%) regions to relatively infrequent in the North Central, North East and Central regions (3–11%) (Fig. 3).

The richness and identity of key mammalian food items (≥0.04 kg per scat) in the diet of wild dogs and foxes varied among regions (Figs. 4–5), as did the frequencies of mammalian food items accounting for >10% of records in the diets of wild dogs and foxes (Table 3). However, some species were consistently important in the diet (Figs. 4–5). For example, key food items for wild dogs in the Mallee, Wimmera and Northern Country regions were the introduced European rabbit (*Oryctolagus cuniculus*), native macropods and livestock, whereas in the North Central region key food items were the introduced sambar deer and native mammals (Fig. 4). Similarly, key food items for foxes ranged from introduced herbivores, macropods and livestock in the Mallee and Northern Country regions to native rodents, macropods and possums in East Gippsland (Fig. 5).

**Table 3:** Results of chi-square tests for homogeneity comparing frequencies of the mammalian food items that accounted for >10% of records in scat samples Victoria-wide for each of the wild dog and the fox among nine Victorian regions based on analysis of scats collected during 1983–2014. Sample sizes are provided in Table 1.

Taxa	*χ* ^2^	df	N	p
Wild dog				
European rabbit	285	8	5094	<0.001
Brushtail possum spp.	201	8	5094	<0.001
Black wallaby	217	8	5094	<0.001
Common wombat	153	8	5094	<0.001
Fox				
House mouse	739	8	11028	<0.001
European rabbit	2881	8	11028	<0.001

Birds occurred most frequently in the diet of wild dogs in the Mallee, Wimmera and Central regions, whereas reptiles/amphibians, insects and plant material occurred most frequently in the diet of wild dogs in the Mallee region and, to a lesser extent, in the Wimmera region (Fig. 3). Birds consistently occurred at relatively low frequencies in the diet of foxes (≤11%; Fig. 3). The occurrence of insects in the diet of foxes was variable, with high frequencies in the Mallee region (68%), very low frequencies in the East Gippsland (2%) and North Central (<1%) regions, and intermediate frequencies in other regions (Fig. 3). Reptiles/amphibians and plant material were also recorded more frequently in the diet of foxes in the Mallee region (16 and 21%, respectively) than from other regions (Fig. 3). Invertebrates other than insects were recorded in very low frequencies (≤1%) in all regions for both wild dogs and foxes.

### Mammal size-classes and key mammalian food items consumed by wild dogs and foxes

Medium and large mammals, and to a lesser extent small mammals, were frequently recorded in the diet of wild dogs (Fig. 3). In contrast, large mammals were relatively less frequent in the diet of foxes (Fig. 3). Food items that accounted for >10% of records in wild dog scat samples Victoria-wide were two large species, black wallaby and common wombat (*Vombatus ursinus*), and medium-sized brushtail possum spp. (S1 Table). In terms of biomass intake, key mammalian food items (≥0.04 kg per scat) in the diet of wild dogs across Victoria were the large-sized black wallaby, common wombat, sambar deer and cattle, and the medium-sized European rabbit and brushtail possum spp. (Fig. 4). Additional key mammalian food items for wild dogs that occurred in select regions only were the large western grey kangaroo, eastern grey kangaroo, sheep, and goat, the medium common ringtail possum (*Pseudocheirus peregrinus*) and the small-sized bush rat (*Rattus fuscipes*) (Fig. 4). By contrast, food items that accounted for >10% of records in the diet of foxes were the small-sized house mouse (*Mus musculus*) and the medium-sized European rabbit (S1 and S2 Tables). Similarly, in terms of biomass, the key mammalian food items of foxes for all regions combined included small (house mouse), medium (European rabbit) and large (sheep) species (Fig. 5). Additional key mammalian food items for foxes that occurred in select regions only were the large western grey kangaroo, eastern grey kangaroo, swamp wallaby, sambar deer and cattle, the medium common ringtail possum and brushtail possum spp., and the small bush rat, dusky antechinus (*Antechinus swainsonii*) and broad-toothed rat (*Mastacomys fuscus*) (Fig. 5).

### Large introduced herbivores in the diets of wild dogs and foxes

Ten large introduced mammal species were recorded in the diets of wild dogs and foxes in Victoria, including two livestock species (S1 and S2 Tables). Sheep was the most frequently recorded large introduced herbivore species in the diet of both wild dogs and foxes, but this species was more than twice as frequent in the diet of foxes (S1 and S2 Tables). Cattle were less frequently recorded than sheep in the diets of both carnivores, but was 1.9 times more frequent in the diet of wild dogs than foxes (S1 and S2 Tables). When data for all regions were combined, the biomass of sheep consumed by foxes was greater than the biomass of sheep consumed by wild dogs, while the reverse was true for cattle (Table 4). Similarly, when data for all regions were combined, sheep were key mammalian food items (≥0.04 kg per scat) for foxes (Fig. 5) while cattle were a key food item for wild dogs (Fig. 4).

**Table 4:** Mass (kg per scat) of large herbivore species in the diets of wild dogs and foxes in Victorian regions, based on analysis of scats collected during 1983–2014. Sample sizes are provided in Table 1.

	kg per scat									
Taxa	All regions	Mallee	Wimmera	Northern	North	East	West/South	Central	North	South
	regions			Country	East	Gippsland	Gippsland		Central	West
Wild dog										
Sheep	0.0314	0.1414	0.1313	0.0418	0.0121	0.0129	0.0022	0.0204	0	0.0593
Cattle	0.0483	0.0533	0	0.1654	0.0423	0.0448	0.0437	0.0809	0	0.1956
Goat	0.0080	0.0225	0	0	0.0028	0.0022	0.0061	0.0163	0.0042	0.0992
Sambar deer	0.0398	0	0	0	0.0648	0	0.0177	0.0358	0.1747	0
Hog deer	0.0002	0	0	0	0	0	0	0	0	0
Red deer	0	0	0	0	0	0	0	0	0	0
Undetermined deer species	0.0038	0	0	0	0.0056	0	0.0108	0.0042	0.007495	0
Horse	0.0008	0	0	0	0	0	0	0.0123	0	0
Pig	0.0011	0	0	0	0.0009	0.0011	0	0.0034	0	0.0133
Eastern grey kangaroo	0.0089	0	0	0.0549	0.0084	0.0142	0.0039	0.0224	0	0
Western grey kangaroo	0.0149	0.0981	0.0615	0	0	0	0	0	0	0.0540
Red kangaroo	0.0023	0.0165	0	0	0	0	0	0	0	0
Fox										
Sheep	0.0664	0.1185	0.1070	0.1500	0.0072	0.0035	0.0020	0.0409	0	0.0209
Cattle	0.0255	0.0325	0.0193	0.0743	0.0124	0.0274	0.0159	0.0463	0	0
Goat	0.0049	0.0084	0.0133	0	0.0011	0	0.0018	0.0027	0	0.003
Sambar deer	0.0045	0	0	0	0.0088	0	0.0481	0	0	0
Hog deer	0.0003	0	0	0	0	0	0.0078	0	0	0
Red deer	0.0002	0	0.0152	0	0	0	0	0	0	0
Undetermined deer species	0.0005	0	0.0079	0	0.0005	0	0.0033	0.0024	0	0
Horse	0	0	0	0	0	0	0	0	0	0
Pig	0.0002	0	0	0	0.0004	0	0	0.0020	0	0
Eastern grey kangaroo	0.0031	0	0.0213	0.0575	0.0060	0.0030	0.0018	0.0038	0	0.0052
Western grey kangaroo	0.0230	0.0417	0.0417	0	0	0	0	0	0	0.0791
Red kangaroo	0.0066	0.0136	0	0	0	0	0	0	0	0

Sambar deer was the only wild large introduced herbivore that was a key food item for wild dogs when data for all regions were pooled (Fig. 4), and was most frequent in diets of wild dogs in the North Central (10%) and North East regions (4%) (S1 Table). Overall, sambar deer comprised approximately 10% of the biomass in fox compared to wild dog diets (Table 4). Sambar deer were recorded in the diets of foxes in West and South Gippsland (2.6%) and North East (0.5%) regions (S2 Table). Hog deer (*Axis porcinus*) were recorded in the diets of both wild dogs and foxes in low frequencies (0.13% in West and South Gippsland/East Gippsland in the diet of wild dogs [data from the two Gippsland regions were pooled because the locations for wild dog scats containing hog deer were too imprecise to assign them to only one region], and 1.09% in West and South Gippsland only in the diet of foxes) and red deer (*Cervus elaphus scoticus*) were recorded only in the diet of foxes, at even lower frequencies, and only in the Wimmera region (S1 and S2 Tables).

The biomass of goat and pig in the diets of wild dogs and foxes was low compared to other large herbivores (≤0.008 kg per scat for wild dogs and ≤0.004 kg per scat for foxes) yet goats were a key mammalian food item for wild dogs in the South West region (Fig. 4). These trends were reflected in frequency of occurrences for goat and pig in the diets of both wild dogs and foxes, which were intermediate and low, respectively, compared to other mammals in the carnivores’ diets (S1 and S2 Tables). Horse was recorded only in the diet of wild dogs in the Central region at low frequencies (S1 Table).

### Large macropods in the diet of wild dogs and foxes

When data from all regions were pooled, biomass intake was greater by wild dogs than foxes for eastern grey kangaroo (Table 4) and eastern wallaroo (0.6 g and 0.2 g per scat, respectively). By contrast, biomass intake of western grey kangaroo and red kangaroo was greater for foxes than wild dogs. The biomass of kangaroo species consumed by wild dogs and foxes varied among regions (Table 4). For example, intake of western grey kangaroo, which occurred in the diets of wild dogs and foxes in the western regions (the Mallee, Wimmera and South West), was highest in the Mallee for wild dogs but highest in the South West by foxes, whereas intake of eastern grey kangaroo was highest in the Northern Country region (Table 4).

### Native Critical Weight Range species in the diets of wild dogs and foxes

**Fig 7:**
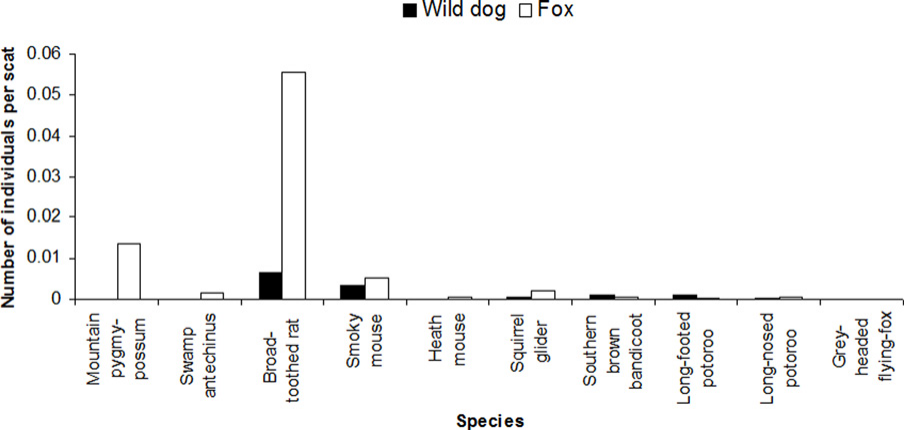
Number of individuals (per scat) of threatened native Critical Weight Range (35–5500 g) mammal species in the diets of wild dogs (*n* = 5148) and foxes (*n* = 11,143) in Victoria, based on analysis of scats collected during 1983–2014.

Of the native mammals recorded in the diets of wild dogs and foxes, small species, most of which fall within the CWR, were numerically dominant for wild dogs (small, 18 ± 6 [mean ± SE] individuals per 1000 scats; medium, 5 ± 2; large, 1 ± 0.6) and foxes (small, 52 ± 14; medium, 3 ± 2; large, 0.01 ± 0.01). Foxes consumed almost twice the number of individuals of species in the CWR than did wild dogs (0.9 and 0.5 individuals per scat, respectively) and 6.1 times the number of threatened CWR species (Fig. 7). In particular, foxes consumed a large number of individuals of the threatened broad-toothed rat and the critically endangered mountain pygmy-possum (*Burramys parvus*) compared to intake of other threatened species in the CWR and compared to intake of any threatened species in the CWR by wild dogs (Fig. 7). Other threatened CWR species in the diets of wild dogs and foxes included small dasyurids, rodents, possum and glider species, potoroos, southern brown bandicoot (*Isoodon obesulus*) and grey-headed flying-fox (*Pteropus poliocephalus*) (S1 and S2 Tables; Fig. 7).

Eleven mammal species of conservation significance listed under state and federal legislation, and/or on the International Union for Conservation of Nature red list were recorded in the diets of wild dogs and 13 mammal and one crustacean species in the diet of foxes (S1 and S2 Tables). Most of these were within the CWR (Fig. 7), but two were smaller (white-footed dunnart (*Sminthopsis leucopus*) and New Holland mouse (*Pseudomys novaehollandiae*)) and two were larger (eastern wallaroo and brush-tailed rock wallaby (*Petrogale penicillata*)). Notably, within the CWR the critically endangered mountain pygmy-possum and the endangered Leadbeater's possum (*Gymnobelideus leadbeateri*) [102] were recorded in the diet of foxes, and the endangered smoky mouse (*Pseudomys fumeus*) [102] was recorded in the diets of both wild dogs and foxes. All listed species of conservation significance in the diet of wild dogs were recorded at frequencies of <0.4% and in the diet of foxes frequencies were generally <0.2% except for broad-toothed rat which was recorded at a frequency of 1.5% (S1 and S2 Tables).

### Intraguild predation/scavenging

Foxes occurred in the diet of wild dogs at low frequency (0.2%) and biomass, but this was double the frequency (0.1%) and 1.5 times the biomass that wild dogs occurred in the diet of foxes (S1 and S2 Tables; Fig. 6). In particular, foxes were consumed by wild dogs at frequencies almost 17-fold greater in the Wimmera region than in all regions combined (S1 and S2 Tables). Wild dog was recorded in the diet of wild dogs, and fox was recorded in the diet of foxes, again at relatively low frequencies compared to other mammalian prey (0.5% and 0.1%, respectively; S1 and S2 Tables). Wild dogs and foxes consumed cats at relatively low and similar frequencies (c. 0.2%; S1 and S2 Tables). Although cat biomass intake by wild dogs in some regions was much greater than that by foxes, Victoria-wide intake of cat by foxes was similar to that by wild dogs (Fig. 6). The frequency of cat in the diet of wild dogs and foxes was relatively great in the Northern Country region (2.27% and 1.02%, respectively; S1 and S2 Tables). The native spot-tailed quoll (*Dasyurus maculatus*) was not recorded in the diets of wild dogs or foxes during this study, but has been recorded in the diet of wild dogs in the literature (S1 Table).

## Discussion

Understanding the diets of sympatric mammalian carnivores is of central importance for ecological theory (niche partitioning) and for managing their impacts on production and conservation values. Our study revealed substantial overlap in the diets of the larger-bodied wild dog and the smaller-bodied red fox in Victoria. However, the breadth of their diet niches and the extent of diet overlap varied geographically, confirming the opportunistic feeding habits of these carnivores in south-eastern Australia [38, 39, 80]. Relative to wild dogs, the diet of foxes consisted of less mammals and more reptiles, insects and plant material. Niche partitioning also appeared to occur on the basis of mammalian prey size: wild dogs more regularly consumed larger mammals than foxes. In particular, the biomass of most wild, large introduced herbivores was greater in the diet of wild dogs than foxes, although the biomass of some domestic livestock and large native herbivore species was greater in the diet of foxes. Foxes consumed more individuals of threatened native mammal species in the CWR than did wild dogs. The occurrence of intraguild predation was asymmetrical; the wild dog consumed greater biomass of the smaller fox than *vice versa*. Hence, four of our six predictions were supported, and we found partial support for our remaining two predictions.

### Diets of wild dogs and red foxes in Victoria

The diets of wild dogs and foxes in Victoria consisted of similar numbers of mammal species (57 and 62, respectively). Scat analysis can be biased towards identification of mammalian prey, for example due to digestion of soft-bodied invertebrates [76, 103], high fragmentation of invertebrate exoskeleton and bird remains [104], lack of or poor diagnostic features for most bird and herpetofauna remains, and sampling biases [105]. Nonetheless, the diet of wild dogs in Victoria was composed of a greater proportion of mammals than was the diet of foxes, consistent with previous work [45, 46, 49, 80, 106, 107].

Although insects may be eaten in large quantities by young wild dogs [108], we found that foxes ate insects and other non-mammalian prey such as birds and reptiles more frequently than did wild dogs and this was reflected in their greater dietary breadth across Victoria, a finding consistent with other studies in south-eastern Australia [46] as well as international studies highlighting the broad trophic spectra of the diet of foxes [109]. This partly explains the success of foxes as one of the world’s most widespread mammalian species. Although foxes frequently consumed mammalian food items, the range of body sizes was nested within the range recorded in the diet of wild dogs. However, this effect was not as pronounced as in some other carnivore guilds [18], perhaps because we were unable to separate diet items that were hunted from those that were scavenged.

### Regional variation in the diets of wild dogs and foxes

As predicted, the diets of wild dogs and foxes in Victoria varied geographically. The greatest variation was between regions that included large tracts of semi-arid rangeland (i.e., Mallee and Wimmera) and wet montane forest (e.g., North East and East Gippsland). In general, mammals were more prevalent in the diets of wild dogs and foxes in the wetter, more montane regions of eastern Victoria than in the more arid regions in western Victoria where non-mammalian food groups including reptiles, insects and plant material, were recorded in higher frequencies in the diets of both carnivores. The suites of key mammalian food items varied regionally, supporting observations that intake of native species is greatest in regions with a large proportion of native forest [110] and alpine areas [106] and conversely that the intake of introduced European rabbits and livestock is greatest in regions dominated by agricultural habitat [49, 111, 112]. The wide regional variation observed in the diet of wild dogs and foxes likely reflects opportunistic and flexible foraging related to the availability of food resources [38, 39, 80, 109, 110, 113–115] which vary according to factors such as habitat [80, 116, 117] and season [113]. In particular, some regional variation in diets reflect the limited distributions of some prey species in Victoria, for example intake of western grey kangaroo only in the three westernmost regions where this species occurs. Anthropogenic food sources may also be important in some areas [38, 118]. Regional diet estimates were potentially biased by scat collection being concentrated in particular habitats and/or seasons. However, we considered that such biases were likely to be low given that our sample sizes were large, except for wild dogs in the Wimmera and South West regions and wild dogs and foxes in the Northern Country region (Table 1), and samples were collected from numerous sites throughout each region and represented all seasons.

### Diet overlap

Ecological separation of sympatric carnivore species around the world is primarily related to dietary differences [119, 120], and, where diet overlap occurs, resources are generally partitioned along spatial or temporal niche dimensions [15, 121]. Diet overlap between wild dogs and foxes Victoria-wide was intermediate compared to that recorded elsewhere in Australia [47, 79, 107, 122]. However, lower levels of overlap than recorded in other parts of Australia occurred in the North Central region and greater levels of overlap were recorded in the Northern Country region. This indicates high potential for interspecific competition if resources are limited [15, 46, 47, 107], although sample sizes in the Northern Country region may not have been large enough to provide accurate diet estimates [123]. Despite diet overlap, our results support the hypothesis that niche partitioning between wild dogs and foxes occurs on the basis of mammalian prey size [46, 47]. Further, while the size-range of mammals in the diet of foxes was nested within that of wild dogs, foxes exhibited a broader diet, indicating dietary separation based on the greater use of non-mammalian food items by the fox.

### Large mammals in the diets of wild dogs and foxes

In contrast to foxes, wild dogs may co-operatively hunt to kill prey much larger than themselves [113, 115, 124]. It was therefore unsurprising that the diet of wild dogs was composed more of large mammalian prey than the diet of foxes. Two limitations of carnivore diet analysis likely explain the inclusion of large species among the key mammalian food items of foxes. First, hair analysis does not readily allow differentiation between young animals and adults [81], nor between sexes. This is an important limitation because young animals and smaller-bodied females in sexually dimorphic species may comprise the bulk of individuals of large species consumed [80], and the use of mean adult body mass to estimate biomass may be problematic in estimating diet composition given the unknown ages and sexes of the individuals consumed [27]. Second, large prey in fox scats are likely to have been scavenged as carrion [106, 125]. Nonetheless, whether hunted or scavenged, large prey contributed substantially to the diet of foxes in Victoria.

### Large introduced herbivores in the diets of wild dogs and foxes

Wild dogs hunt domestic livestock, with sheep and goats being particularly vulnerable [126]. However, there was mixed support for our prediction that the diet of wild dogs would be composed of more livestock species than that of foxes: sheep occurred at greater frequencies in the diet of foxes than in the diet of wild dogs, whereas cattle were recorded in greater frequencies in the diet of wild dogs. Although calves are at risk of wild dog predation [127], cattle are less vulnerable than sheep due to inherent size and behavioural differences [126]. However, cattle are eaten by wild dogs when the availability of smaller prey is low [113], largely as carrion [128]. The occurrence of sheep among the key mammalian food items of foxes probably results largely from lamb predation [8, 69], but may also reflect scavenging of carcasses that have not been disposed of. Cattle in fox diet is likely to be scavenged [126], although foxes have been implicated in the deaths of calves and older cattle [38].

Overall, our findings support our prediction that use of large introduced herbivores is greater by wild dogs than by foxes. Sambar deer were a key mammalian food item in the diet of wild dogs, and to a lesser extent foxes, in several regions where this species is now common [129, 130], which has not previously been documented. Wild dogs may have been hunting and killing sambar deer, particularly calves, but given the large size of sambar deer (110–240 kg [82]) consumption of this food item by wild dogs and foxes could reflect scavenging [125]. The greater use of sambar deer by wild dogs may be due to the greater jaw size and strength enabling wild dogs to better access carcasses. However, the use of this food resource by foxes may be underestimated if they are scavenging from opened carcasses and not ingesting hair.

Large introduced herbivores other than sambar deer were recorded in the diets of wild dogs and foxes in relatively low frequencies. This result may reflect the low densities and/or limited distributions of some species in Victoria [131], and the low susceptibility of other species to wild dog and/or fox predation.

### Large macropods in the diets of wild dogs and foxes

Our prediction that large native herbivores would be more common in the diet of wild dogs than in the diet of foxes was supported for only two of the four large macropod species in Victoria, and kangaroos were key mammalian food items for both wild dogs and foxes. Macropods of all age-sex classes are actively hunted by wild dogs [80], whereas foxes actively hunt young kangaroos, although they may also chase adult female eastern grey kangaroos so that they eject their pouch young [132, 133]. Hence, it is likely that much of the macropod material in the diet of foxes was scavenged [38, 110]. In the absence of information regarding use of carrion, we cannot determine whether predation on large macropods is greater by wild dogs than by foxes.

### Other key mammalian food items

Our study indicates that wild dogs and foxes eat a wide range of mammals in Victoria, but the majority of their diets are composed of a relatively small proportion of these species, consistent with previous studies [39, 76, 80, 81, 134, 135]. In addition to the large introduced herbivores and large macropods that were key food items for wild dogs and foxes, other medium- and large-sized native mammal species were important components of the diet of wild dogs in this and previous studies [49, 128, 136], and several small-, medium- and large-sized native and introduced mammal species were important in the diet of foxes in this and previous studies [49, 76, 81, 110, 137]. The prevalence of arboreal species in the diets of both wild dogs and foxes may be explained by the descent to the ground of these species when the overstorey is discontinuous [138, 139].

Importantly, our analysis indicates that the European rabbit is a key food item for wild dogs in the Mallee, Wimmera, Northern Country, North East, Central and South West regions. The European rabbit can be an important component of the diet of wild dogs in arid Australia [113, 116], but has not previously been shown to be an important food in south-eastern Australia. This finding most likely reflects more widespread sampling in our study than has occurred during previous work on the diets of wild dogs in south-eastern Australia, much of which has been focused in forested habitats [134]. The European rabbit is a key prey species for foxes internationally [109, 140] and throughout their Australian range [40, 110, 141, 142], hence it was unsurprising that this species was among the key mammalian food items for foxes in six of the nine Victorian regions.

### Native Critical Weight Range species in the diet of wild dogs and foxes

Our prediction that the diet of foxes contained more native species in the CWR than did the diet of wild dogs was supported: foxes consumed 1.8 times the number of individuals of species in the CWR than wild dogs and 6.1 times the number of individuals of threatened species in the CWR. Furthermore, a slightly greater number of mammal species of conservation significance, most of which were within the CWR, were recorded in the diet of foxes. Our results are supported by previous work suggesting that foxes pose a greater risk to mammals within the CWR than do wild dogs [45, 79], particularly as they typically occur at higher densities [54], but that wild dogs also have the potential to impact on populations of small prey [26, 143]. Of the native CWR species, foxes appear to prey heavily on the threatened broad-toothed rat, and to a lesser extent on the critically endangered mountain pygmy-possum. Previous studies have identified the broad-toothed rat as a food item of foxes [48, 50] and relatively high frequencies of this species have been recorded in the diet of foxes in Central Victoria [76]. The frequencies of these species in wild dog and fox diets may have been overestimated if sampling was targeted within their ranges, or aimed to detect small mammal species. However, samples were collated from numerous sources and hence such biases were likely minimal. Moderate to heavy predation on broad-toothed rat even in localised areas, particularly in the North Central region where it was a key food item for foxes, may represent a threat to this species, particularly because it is selectively preyed upon by foxes [144]. Although other species of conservation significance were recorded in carnivore diets at low frequencies, this does not necessarily indicate low risk. Occurrence may be a function of availability and in isolation does not provide information on diet preferences and therefore how diet may change as resource availability changes [26, 143, 145]. Even low levels of predation by wild dogs and/or foxes may be important for rare species [146].

### Intraguild predation/scavenging

Carnivores may scavenge carrion of other smaller, larger, or their own species [22, 23]. We therefore cannot discount the records of carnivores in the diets of wild dogs and foxes as being from scavenging rather than from intraguild predation and cannibalism. However, larger carnivores are known to kill smaller competitors [15]. Although the frequency and biomass of fox in the diet of wild dogs in Victoria was low, it was approximately double that of wild dog in the diet of foxes. This finding is consistent with records of wild dogs killing foxes in Australia, but not *vice versa* [107]. The occurrence of intraguild predation between wild dogs and foxes therefore appears to be asymmetrical [21, 35, 147]. Despite foxes being renowned killers of other carnivores [21], the larger wild dog asserts predatory and competitive dominance over foxes in Australia [35]. Foxes exhibit behavioural avoidance of wild dogs [125] and wild dogs suppress foxes due to direct predation and competition [45, 147–149]. Our results indicate a potential for suppression of foxes by wild dogs in Victoria, particularly in the Wimmera, Mallee and North Central regions where the occurrence of fox in the diet of wild dogs was highest.

Wild dogs and foxes consumed cats at similarly low frequencies in Victoria. Consumption of cats by wild dogs [134] and foxes has been previously recorded [110, 122, 150] and there is evidence to suggest that both carnivores suppress feral cat populations in tropical, arid and semi-arid environments [142, 148, 151]. Intraguild predation of cats may indicate the potential for suppression of cats, particularly by wild dogs, and particularly in the Northern Country region where the frequency of cat in the diet of both carnivores was relatively high. However, it is unclear what impact the low frequency of intraguild predation has on feral cat populations. The incidence of interspecific killing may be underestimated by diet studies because animals killed are not always consumed [47, 147].

Cannibalism and scavenging on dead conspecifics has been documented in wild dogs [49, 116, 152] and foxes [48]. In our study, wild dog was recorded in the diet of wild dogs and fox was recorded in the diet of foxes. However, the low frequencies suggest that cannibalism is infrequent in this guild.

### Implications for management

Changes in the availability of foods that frequently occur in the diet may have implications for the abundance and/or impacts of carnivores [153–155]. Wild dogs consumed more large mammals than did foxes, and the introduced sambar deer was an important component of wild dog diet in several regions and of fox diet in one region. The introduced European rabbit was often a key food item for both foxes and wild dogs. Management actions that increase the short-term availability of these foods may lead to increased food availability for wild dogs and foxes. Conversely, management actions that alter the abundances of fox and wild dog populations should consider the potential for release of prey populations [34].

Foxes rather than wild dogs are thought to be a major cause of the decline of Australia’s CWR mammals [45, 66, 79] and substantial fox control is conducted within Victoria to protect native mammals [72]. Two findings from this study suggest that foxes are more important predators of CWR mammals in Victoria than wild dogs. First, wild dogs generally consumed larger mammalian food items than foxes. Second, although numbers of threatened native mammal species recorded in the diets of wild dogs and foxes were similar, foxes consumed a greater number of individuals of native mammal species in the CWR than did wild dogs.

Finally, large geographic variation in the diets of wild dogs and foxes indicates that a wide variety of trophic interactions are possible within the State of Victoria [35]. This geographic variation is a function of the high diversity of ecosystems (i.e., spanning semi-arid, mesic and alpine) in Victoria. Our regional checklists of taxa (and their frequency) recorded in the diets of wild dogs and foxes within the nine Victorian regions will assist managers and researchers wishing to understand the potential impacts of policy or management decisions on wild dogs and/or foxes and/or the food resources they interact with.

## Supporting Information

S1 Appendix Comparison of three measures used to estimate the diets of wild dogs and red foxes. (DOCX)

S1 Table Checklist of taxa recorded in the diet of dingoes/wild dogs in Victoria. (DOCX)

S2 Table Checklist of taxa recorded in the diet of foxes in Victoria. (DOCX)
