# Correction: Interspecific and Geographic Variation in the Diets of Sympatric Carnivores: Dingoes/Wild Dogs and Red Foxes in South-Eastern Australia

**DOI:** 10.1371/journal.pone.0130241

**Published:** 2015-06-04

**Authors:** Naomi E. Davis, David M. Forsyth, Barbara Triggs, Charlie Pascoe, Joe Benshemesh, Alan Robley, Jenny Lawrence, Euan G. Ritchie, Dale G. Nimmo, Lindy F. Lumsden

S1 Table is incorrectly labeled as S2 Table in the Supporting Information. The complete, correct title is: S1 Table. Checklist of taxa recorded in the diet of dingoes/wild dogs in Victoria. There are also errors in S1 Table, “Checklist of taxa recorded in the diet of dingoes/wild dogs in Victoria”. Please see the corrected S1 Table here.

S2 Table is incorrectly labeled as Table S3 in the Supporting Information. The complete, correct title is: S2 Table. Checklist of taxa recorded in the diet of foxes in Victoria. There are also errors in S2 Table, “Checklist of taxa recorded in the diet of foxes in Victoria”. Please see the corrected S2 Table here.

## Supporting Information

S1 Table Checklist of taxa recorded in the diet of dingoes/wild dogs in Victoria. (DOCX)

S2 Table Checklist of taxa recorded in the diet of foxes in Victoria. (DOCX)
