# Supplementary material for: Interspecific and Geographic Variation in the Diets of Sympatric Carnivores: Dingoes/Wild Dogs and Red Foxes in South-Eastern Australia
Source: PLoS One. 2015 Mar 19;10(3):e0120975. doi: 10.1371/journal.pone.0120975 (PMC4366095; doi:10.1371/journal.pone.0120975)
Supplement: S1 Appendix — (DOCX) [file pone.0120975.s001.docx]

**S1 Appendix.** Comparison of three measures used to estimate the diets of wild dogs and red foxes.

We used three metrics to estimate the diets of wild dogs and red foxes: 1. frequency occurrence; 2. relative total biomass; and 3. relative number of individuals of each prey type consumed. Frequency of occurrence was our primary metric. The frequency occurrence, *F*, of a given prey type *i* is deﬁned as the number of samples *N* in which that prey occurs, expressed as a frequency of the total number of samples (or of the number of samples that contained prey) [1,2]:

$$F_{i}=\left(\frac{N_{i}}{N}\right)\times100.$$

However, frequency occurrence underestimates the relative number of small mammalian prey consumed and overestimates their relative biomass in the diet [3]. This occurs because smaller mammals have a higher surface:volume ratio than larger mammals and therefore have relatively more hair per unit mass of flesh [4]. Further, smaller mammals are composed of a relatively greater proportion of indigestible material than larger mammals [5]. It has been suggested that biomass calculation models based on feeding trials reduce these biases and provide the best approximation of the diet of mammalian carnivores [6]. Therefore, we explored the relationship between estimates of mammalian species in the diet based on frequency occurrence, and estimates of biomass and the number of individuals of mammalian species in the diet, estimated from the linear regression model developed by Floyd et al. [5] for wolf (*Canis lupus*) with modiﬁcations by Weaver [3] that ensured it spans a range of prey sizes from snowshoe hare (*Lepus americanus*; 1.16 kg) to adult moose (*Alces alces*; >300 kg):

$$Y=0.439+0.008X,$$

where *Y* is the mass of prey per scat and *X* is the mean mass of an individual of a given prey type. We multiplied *Y* by the number of scats in which each prey type was recorded to estimate the relative total mass of each prey type consumed per region, and then divided this value by the body mass of each species to estimate the relative number of individuals of each prey type consumed per region, as represented by the scat sample.

We therefore evaluated which of the three metrics (i.e. frequency occurrence, relative total biomass, and relative number of individuals of each prey type consumed) was most appropriate for examining each of our predictions. We examined the relationship between the three metrics graphically for all mammalian prey items identified to species level in wild dog and fox diets for the nine Victorian regions. A large number of mammalian prey items occurred in the diets of wild dogs and foxes in Victoria (57 and 62, respectively) and relationships between the three diet metrics for mammals of different size-classes were similar for wild dogs and foxes. Hence to simplify demonstration of these relationships, we present data for wild dog diet only, and we present comparisons of two representative mammalian prey items from each size class: small- (0.001–0.499 kg), medium- (0.5–6.999 kg) and large-sized (≥7 kg) mammalian prey.

Our results (Fig A), illustrate our general finding that the relative biomass and number of individuals tracked trends in frequency occurrence of species in the diet. However, counts of individuals were skewed by relatively high numerical intake of small species, and comparisons of the relative biomass of species consumed emphasised the high biomass intake for large species [3,4]. We considered the number of individuals of each species consumed (standardised per scat) to be the most sensible metric for evaluating the potential impacts of wild dogs and foxes on species of conservation significance (mostly small- and medium-sized species) because in this instance our primary aim was to determine potential impacts on prey species. However, estimates of the number of individuals consumed emphasises small species. We therefore selected relative biomass as the most appropriate metric to evaluate regional and interspecific differences in the use of key prey species because this measure is likely to better reflect the relative importance of different species as food sources for wild dogs and foxes in Victoria.

**Fig A.** Three metrics of wild dog diet in Victoria. Data are *n* = 5875 wild dog scats samples collected in Victoria.

**References**

1. Corbett LK. Contributions to the biology of dingoes (Camivora: Canidae) in Victoria. M. Sc. Thesis, Monash University. 1974. Available: http://trove.nla.gov.au/version/27100205.

2. Amundsen PA, Gabler HM, Staldvik FJ. A new approach to graphical analysis of feeding strategy from stomach contents data—modiﬁcation of the Costello (1990) method. J Fish Biol. 1996;48: 607-614.

3. Weaver JL. Refining the equation for interpreting prey occurrence in gray wolf scats. J Wildl Manage. 1993;57: 534-538.

4. Mech LD. The wolf; the ecology and behaviour of an endangered species. New York: Natural History Press, Doubleday; 1970.

5. Floyd TJ. Relating wolf scat content to prey consumed. J Wildl Manage. 1978;42: 528-532.

6. Klare U, Kalmer JF, MacDonald DW. A comparison of different scat-analysis methods for determining carnivore diet. Mamm Rev. 2011;41: 294-312.
