# Supplementary material for: Interspecific and Geographic Variation in the Diets of Sympatric Carnivores: Dingoes/Wild Dogs and Red Foxes in South-Eastern Australia
Source: PLoS One. 2015 Mar 19;10(3):e0120975. doi: 10.1371/journal.pone.0120975 (PMC4366095; doi:10.1371/journal.pone.0120975)
Supplement: S1 Table — (DOCX) [file pone.0120975.s002.docx]

**S2 Table.** Checklist of taxa recorded in the diet of dingoes/wild dogs in Victoria.**

| Food item | Family (Order for insects) | Common name | Scientific name | | All regions | | Mallee | | Wimmera | | Northern Country | | North East | | East G | | West/South G | | Central | | North Central | | South West | |
| --- | --- | --- | --- | --- | --- | --- | --- | --- | --- | --- | --- | --- | --- | --- | --- | --- | --- | --- | --- | --- | --- | --- | --- | --- |
|  |  |  |  | | P | % | P | % | P | % | P | % | P | % | P | % | P | % | P | % | P | % | P | % |
| Mammal | Acrobatidae | Feathertail glider | *Acrobates pygmaeus* | | 1 | 0 |  | 0 |  | 0 |  | 0 |  | 0 | 1 | 0 |  | 0 |  | 0 |  | 0 |  | 0 |
|  | Bovidae | Sheep | *Ovis aries** | | 1 | 3.42 | 1 | 15.38 | 1 | 14.29 | 1 | 4.55 | 1 | 1.31 | 1 | 1.41 | 1 | 0.24 | 1 | 2.22 |  | 0 | 1 | 6.45 |
|  |  | Cattle | *Bos taurus** | | 1 | 1.33 | 1 | 1.47 |  | 0 | 1 | 4.55 | 1 | 0.61 | 1 | 1.23 | 1 | 1.20 | 1 | 2.22 |  | 0 | 1 | 5.38 |
|  | Burramyidae | Eastern pygmy-possum | *Cercartetus nanus*^ƹ^ | | 1 | 0.12 |  | 0 |  | 0 |  | 0 | 1 | 0.22 | 1 | 0.09 |  | 0 |  | 0 |  | 0 |  | 0 |
|  |  | Undetermined pygmy-possum | *Cercartetus* sp. | | 1 | 0.02 | 1 | 0.12 |  | 0 |  | 0 |  | 0 |  | 0 |  | 0 |  | 0 |  | 0 |  | 0 |
|  | Canidae | Dingo/dog | *Canis dingo/familiaris** | | 1 | 0.46 | 1 | 0.61 |  | 0 |  | 0 | 1 | 0.15 | 1 | 0.44 | 1 | 0.48 | 1 | 1.67 | 1 | 0.50 | 1 | 1.08 |
|  |  | Red fox | *Vulpes vulpes** | | 1 | 0.17 | 1 | 0.49 | 1 | 2.86 |  | 0 | 1 | 0.07 | 1 | 0.09 |  | 0 | 1 | 0.28 | 1 | 0.50 |  | 0 |
|  | Caprinae | Goat | *Capra hircus**^,ƹ^ | | 1 | 0.95 | 1 | 2.69 |  | 0 |  | 0 | 1 | 0.34 | 1 | 0.26 | 1 | 0.72 | 1 | 1.94 | 1 | 0.50 | 1 | 11.83 |
|  | Cervidae | Sambar deer | *Rusa unicolor** | | 1 | 2.16 |  | 0 |  | 0 |  | 0 | 1 | 3.52 | 1^ǂ^ | 0.26 | 1 | 0.96 | 1 | 1.94 | 1 | 9.50 |  | 0 |
|  |  | Hog deer | *Axis porcinus**^,ƹ^ | | 1 | 0.03 |  | 0 |  | 0 |  | 0 |  | 0 |  | 0 |  | 0 |  | 0 |  | 0 |  | 0 |
|  |  | Undetermined deer* | | | 1 | 0.26 |  | 0 |  | 0 |  | 0 | 1 | 0.37 | 1 | 0.26 | 1 | 0.72 | 1 | 0.28 | 1 | 0.50 |  | 0 |
|  | Dasyuridae | Brown antechinus | *Antechinus stuartii* | | 1 | 0.36 |  | 0 |  | 0 |  | 0 | 1 | 0.26 | 1 | 0.70 | 1 | 1.20 | 1 | 0.28 | 1 | 0 |  | 0 |
|  |  | Dusky antechinus | *Antechinus swainsonii* | | 1 | 1.86 |  | 0 |  | 0 |  | 0 | 1 | 2.02 | 1 | 2.55 | 1 | 4.09 | 1 | 0.56 | 1 | 3.00 |  | 0 |
|  |  | Agile antechinus | *Antechinus agilis*^ƹ^ | | 1 | 0.07 |  | 0 |  | 0 |  | 0 | 1 | 0.07 | 1 | 0.18 |  | 0 |  | 0 |  | 0 |  | 0 |
|  |  | Yellow-footed antechinus | *Antechinus flavipes*^ƹ^ | | 1 | 0 |  | 0 |  | 0 |  | 0 |  | 0 |  | 0 |  | 0 |  | 0 |  | 0 |  | 0 |
|  |  | Undetermined antechinus | *Antechinus* sp. | | 1 | 0.14 |  | 0 |  | 0 |  | 0 | 1 | 0.11 | 1 | 0.18 | 1 | 0.24 |  | 0 | 1 | 0.50 |  | 0 |
|  |  | Spot-tailed quoll | *Dasyurus maculatus*^€,£,∫^ | | 1 | 0 |  | 0 |  | 0 |  | 0 |  | 0 | 1 | 0 |  | 0 |  | 0 |  | 0 |  | 0 |
|  |  | Mallee ningaui | *Ningaui yvonneae*^ƹ^ | | 1 | 0.07 | 1 | 0.49 |  | 0 |  | 0 |  | 0 |  | 0 |  | 0 |  | 0 |  | 0 |  | 0 |
|  |  | Common dunnart | *Sminthopsis murina*^ƹ^ | | 1 | 0.03 | 1 | 0.12 |  | 0 |  | 0 | 1 | 0 |  | 0 |  | 0 | 1 | 0.28 |  | 0 |  | 0 |
|  |  | Fat-tailed dunnart | *Sminthopsis crassicaudata*^ƹ^ | | 1 | 0.02 |  | 0 | 1 | 2.86 |  | 0 |  | 0 |  | 0 |  | 0 |  | 0 |  | 0 |  | 0 |
|  |  | Undetermined dunnart | *Sminthopsis* sp. | | 1 | 0.10 | 1 | 0.73 |  | 0 |  | 0 |  | 0 |  | 0 |  | 0 |  | 0 |  | 0 |  | 0 |
|  | Equidae | Horse | *Equus caballus** | | 1 | 0.02 |  | 0 |  | 0 |  | 0 | 1 | 0 | 1 | 0 |  | 0 | 1 | 0.28 |  | 0 |  | 0 |
| Table S1 (cont.) | | | | | | | | | | | | | | | | | | | | | | | | |
| Food item | Family (Order for insects) | Common name | Scientific name | | All regions | | Mallee | | Wimmera | | Northern Country | | North East | | East G | | West/South G | | Central | | North Central | | South West | |
|  |  |  |  | | P | % | P | % | P | % | P | % | P | % | P | % | P | % | P | % | P | % | P | % |
|  | Felidae | Cat | *Felis catus** | | 1 | 0.14 |  | 0 |  | 0 | 1 | 2.27 | 1 | 0.11 | 1 | 0.09 |  |  | 1 | 0.56 |  | 0 |  | 0 |
|  | Hominidae | Human | *Homo sapiens** | | 1 | 0.03 |  | 0 |  | 0 |  | 0 |  | 0 | 1 | 0 |  | 0 | 1 | 0.56 |  | 0 |  | 0 |
|  | Leporidae | European rabbit | *Oryctolagus cuniculus** | | 1 | 11.95 | 1 | 17.83 | 1 | 11.43 | 1 | 25.00 | 1 | 12.07 | 1 | 4.84 | 1 | 5.53 | 1 | 24.72 | 1 | 3.50 | 1 | 37.63 |
|  |  | Brown hare | *Lepus capensis**^,ƹ^ | | 1 | 0.31 |  | 0 |  | 0 |  | 0 | 1 | 0.52 |  | 0 |  | 0 | 1 | 0.83 | 1 | 0.50 |  | 0 |
|  |  | Undetermined lagomorph* | | | 1 | 0.78 |  | 0 |  | 0 |  | 0 | 1 | 0.94 | 1 | 0.18 |  |  | 1 | 1.39 | 1 | 6.50 |  | 0 |
|  | Macropodidae | Red-necked wallaby | *Macropus rufogriseus* | | 1 | 0.26 |  | 0 |  | 0 |  | 0 | 1 | 0 | 1 | 1.32 |  | 0 |  | 0 |  | 0 |  | 0 |
|  |  | Black wallaby | *Wallabia bicolor* | | 1 | 24.68 | 1 | 0.73 | 1 | 28.57 | 1 | 15.91 | 1 | 29.01 | 1 | 36.06 | 1 | 26.70 | 1 | 11.94 | 1 | 32.00 | 1 | 7.53 |
|  |  | Black wallaby or Red-necked wallaby | *W. bicolor* or *M. rufogriseus* | | 1 | 0.02 |  | 0 |  | 0 |  | 0 | 1 | 0 | 1 | 0.09 |  | 0 |  | 0 |  | 0 |  | 0 |
|  |  | Eastern grey kangaroo | *Macropus giganteus* | | 1 | 1.11 |  | 0 |  | 0 | 1 | 6.82 | 1 | 1.05 | 1 | 1.76 | 1 | 0.48 | 1 | 2.78 |  | 0 |  | 0 |
|  |  | Western grey kangaroo | *Macropus fuliginosus*^ƹ^ | | 1 | 2.08 | 1 | 13.68 | 1 | 8.57 |  | 0 |  | 0 |  | 0 |  | 0 |  | 0 |  | 0 | 1 | 7.53 |
|  |  | Red kangaroo | *Macropus rufus*^ƹ^ | | 1 | 0.29 | 1 | 2.08 |  | 0 |  | 0 |  | 0 |  | 0 |  | 0 |  | 0 |  | 0 |  | 0 |
|  |  | Eastern wallaroo | *Macropus robustus robustus*^€,Ω^ | | 1 | 0.10 |  | 0 |  | 0 |  | 0 | 1 | 0.22 | 1 | 0 |  | 0 |  | 0 |  | 0 |  | 0 |
|  |  | Undetermined kangaroo or wallaby | *Macropus* sp. | | 1 | 2.40 | 1 | 14.77 |  | 0 | 1 | 2.27 | 1 | 0.11 | 1 | 1.14 |  | 0 | 1 | 0.83 |  | 0 |  | 0 |
|  |  | Brush-tailed rock-wallaby | *Petrogale penicillata*^€,≠,∫,ƹ^ | | 1 | 0.02 |  | 0 |  | 0 |  | 0 |  | 0 | 1 | 0.09 |  | 0 |  | 0 |  | 0 |  | 0 |
|  |  | Undetermined macropod | | | 1 | 0.54 | 1 | 0.73 |  | 0 | 1 | 4.55 | 1 | 0.30 | 1 | 0.79 | 1 | 0.24 | 1 | 1.67 |  | 0 |  | 0 |
|  | Muridae | Water rat | *Hydromys chrysogaster* | | 1 | 0.15 |  | 0 |  | 0 |  | 0 | 1 | 0 | 1 | 0.44 | 1 | 0.24 | 1 | 0.56 |  | 0 | 1 | 1.08 |
|  |  | Broad-toothed rat | *Mastacomys fuscus*^€,∫^ | | 1 | 0.19 |  | 0 |  | 0 |  | 0 | 1 | 0.19 | 1 | 0.09 | 1 | 0.96 | 1 | 0.28 |  | 0 |  | 0 |
|  |  | House mouse | *Mus musculus** | | 1 | 0.85 | 1 | 5.71 | 1 | 4.52 |  | 0 | 1 | 0.07 | 1 | 0.18 |  | 0 | 1 | 0.28 | 1 | 2.50 | 1 | 1.08 |
|  |  | Mitchell's hopping-mouse | *Notomys mitchellii*^ƹ^ | | 1 | 0.26 | 1 | 1.83 |  | 0 |  | 0 |  | 0 |  | 0 |  | 0 |  | 0 |  | 0 |  | 0 |
|  |  | Smoky mouse | *Pseudomys fumeus*^€,£,∏,ƹ^ | | 1 | 0.05 |  | 0 | 1 | 2.86 |  | 0 | 1 | 0.07 |  | 0 |  | 0 |  | 0 |  | 0 |  | 0 |
|  |  | Silky mouse | *Pseudomys apodemoides*^ƹ^ | | 1 | 0.14 | 1 | 0.98 |  | 0 |  | 0 |  | 0 |  | 0 |  | 0 |  | 0 |  | 0 |  | 0 |
| Table S1 (cont.) | | | | | | | | | | | | | | | | | | | | | | | | |
| Food item | Family (Order for insects) | Common name | Scientific name | | All regions | | Mallee | | Wimmera | | Northern Country | | North East | | East G | | West/South G | | Central | | North Central | | South West | |
|  |  |  |  | | P | % | P | % | P | % | P | % | P | % | P | % | P | % | P | % | P | % | P | % |
|  |  | New Holland mouse | *Pseudomys novaehollandiae*^€,≠,ƹ^ | | 1^ǂ^ | 0.02 |  | 0 |  | 0 |  | 0 |  | 0 | 1^ǂ^ | 0.09 |  | 0 |  | 0 |  | 0 |  | 0 |
|  |  | Bush rat | *Rattus fuscipes* | | 1 | 5.70 |  | 0 |  | 0 | 1 | 4.55 | 1 | 6.93 | 1 | 7.83 | 1 | 7.21 | 1 | 0.83 | 1 | 9.00 | 1^ǂ^ | 1.08 |
|  |  | Swamp rat | *Rattus lutreolus* | | 1 | 1.07 |  | 0 |  | 0 |  | 0 | 1 | 0.22 | 1 | 3.08 | 1 | 3.37 | 1 | 1.39 |  | 0 | 1 | 3.23 |
|  |  | Black rat | *Rattus rattus*^ƹ^ | | 1 | 0.54 | 1 | 0.12 |  | 0 |  | 0 | 1 | 0.30 | 1 | 0.62 | 1 | 1.92 | 1 | 2.22 |  | 0 |  | 0 |
|  |  | Undetermined rat | *Rattus* sp. | | 1 | 1.80 |  | 0 |  | 0 |  | 0 | 1 | 1.31 | 1 | 2.99 | 1 | 5.53 | 1 | 1.67 | 1 | 2.50 | 1 | 1.08 |
|  |  | Undetermined rat | *Rattus* sp. or *Mastacomys* sp. | | 1 | 0.02 |  | 0 |  | 0 |  | 0 |  | 0 |  | 0 |  | 0 | 1 | 0.28 |  | 0 |  | 0 |
|  | | Undetermined rodent | |  | 1 | 0.34 | 1 | 0.85 |  | 0 |  | 0 | 1 | 0.15 | 1 | 0.09 |  | 0 | 1 | 0.56 | 1 | 3.00 |  | 0 |
|  | Ornithorhynchidae | Platypus | *Ornithorhynchus anatinus* | | 1 | 0.31 |  | 0 |  | 0 |  | 0 | 1 | 0.22 | 1 | 0.62 | 1 | 0.96 |  | 0 | 1 | 0.50 |  | 0 |
|  | Peramelidae | Long-nosed bandicoot | *Peremeles nasuta* | | 1 | 3.54 |  | 0 |  | 0 |  | 0 | 1 | 4.69 | 1 | 5.80 | 1 | 3.61 |  | 0 |  | 0 |  | 0 |
|  |  | Southern brown bandicoot | *Isoodon obesulus obesulus*^€,£,Ω^ | | 1 | 0.24 |  | 0 |  | 0 |  | 0 | 1 | 0 | 1 | 1.14 |  | 0 | 1 | 0.28 |  | 0 |  | 0 |
|  |  | Undetermined bandicoot | | | 1 | 0.07 |  | 0 |  | 0 |  | 0 | 1 | 0 | 1 | 0.26 |  | 0 |  | 0 |  | 0 |  | 0 |
|  | Petauridae | Yellow-bellied glider | *Petaurus australis* | | 1 | 0.39 |  | 0 |  | 0 |  | 0 | 1 | 0.60 | 1 | 0.53 | 1^ǂ^ | 0.24 |  | 0 |  | 0 |  | 0 |
|  |  | Sugar glider | *Petaurus breviceps* | | 1 | 0.44 |  | 0 |  | 0 | 1^ǂ^ | 4.55 | 1 | 0.45 | 1 | 0.79 | 1 | 0.72 |  | 0 |  | 0 |  | 0 |
|  |  | Squirrel glider | *Petaurus norfolcensis*^€,Ω,ƹ^ | | 1^ǂ^ | 0.03 |  | 0 |  | 0 | 1^ǂ^ | 4.55 |  | 0 |  | 0 |  | 0 |  | 0 |  | 0 |  | 0 |
|  |  | Undetermined glider | *Petaurus* sp. | | 1 | 0.03 |  | 0 |  | 0 | 1 | 4.55 | 1 | 0 |  | 0 |  | 0 |  | 0 |  | 0 |  | 0 |
|  | Phascolarctidae | Koala | *Phascolarctos cinereus* | | 1 | 0.07 |  | 0 |  | 0 |  | 0 | 1 | 0 | 1 | 0.26 |  | 0 | 1 | 0.28 |  | 0 |  | 0 |
|  | Phalangeridae | Common brushtail possum | *Trichosurus vulpecula* | | 1 | 1.60 | 1 | 0.49 |  | 0 |  | 0 | 1 | 2.44 | 1 | 0.26 | 1 | 1.92 | 1 | 2.50 |  | 0 |  | 0 |
|  |  | Mountain brushtail possum | *Trichosurus caninus*^ƹ^ | | 1 | 1.02 |  | 0 |  | 0 |  | 0 | 1 | 1.50 | 1 | 0.88 | 1^ǂ^ | 1.20 | 1 | 1.39 |  | 0 |  | 0 |
|  |  | Undetermined brushtail possum | *Trichosurus* sp. | | 1 | 17.12 | 1 | 0.49 |  | 0 | 1 | 2.27 | 1 | 22.83 | 1 | 16.40 | 1 | 21.90 | 1 | 10.30 | 1 | 28.50 | 1 | 2.15 |
|  | Potoroidae | Long-footed potoroo | *Potorous longipes*^€,£,∏^ | | 1 | 0.39 |  | 0 |  | 0 |  | 0 | 1 | 0.82 | 1 | 0.09 |  | 0 |  | 0 |  | 0 |  | 0 |
|  |  |  |  | |  |  |  |  |  |  |  |  |  |  |  |  |  |  |  |  |  |  |  |  |
| Table S1 (cont.) | | | | | | | | | | | | | | | | | | | | | | | | |
|  |  |  |  | | All regions | | Mallee | | Wimmera | | Northern Country | | North East | | East G | | West/South G | | Central | | North Central | | South West | |
| Food item | Family (Order for insects) | Common name | Scientific name | | P | % | P | % | P | % | P | % | P | % | P | % | P | % | P | % | P | % | P | % |
|  |  | Long-nosed potoroo | *Potorous tridactylus tridactylus*^€,≠,Ω,ƹ^ | | 1 | 0.10 |  | 0 |  | 0 |  | 0 |  | 0 | 1 | 0.53 |  | 0 |  | 0 |  | 0 |  | 0 |
|  |  | Undetermined potoroo | *Potorous* sp. | | 1 | 0.05 |  | 0 |  | 0 |  | 0 |  | 0 | 1 | 0.26 |  | 0 |  | 0 |  | 0 |  | 0 |
|  | Pseudocheiridae | Common ringtail possum | *Pseudocheirus peregrinus* | | 1 | 4.49 | 1 | 0.12 |  | 0 | 1 | 2.27 | 1 | 3.04 | 1 | 5.80 | 1 | 10.10 | 1 | 12.22 | 1 | 8.50 | 1 | 3.23 |
|  |  | Greater glider | *Petauroides volans* | | 1 | 1.29 |  | 0 |  | 0 |  | 0 | 1 | 2.29 | 1 | 0.26 | 1 | 2.16 | 1 | 0.56 | 1 | 0 |  | 0 |
|  | Pseudocheiridae or Petauridae | Undetermined possum/glider | *Petauroides* sp. or *Petaurus* sp. | | 1 | 0.03 |  | 0 |  | 0 |  | 0 | 1 | 0.04 |  | 0 | 1 | 0.24 |  | 0 |  | 0 |  | 0 |
|  | Phalangeridae, Pseudocheiridae or Peauridae | Undetermined possum/glider | *Trichosurus* sp., *P. peregrinus*, *Petaurus* sp. or *Petauroides* sp. | | 1 | 0.12 |  | 0 |  | 0 |  | 0 | 1 | 0.15 |  | 0 |  | 0 | 1 | 0.28 | 1 | 1.00 |  | 0 |
|  | Pteropodidae | Grey-headed flying-fox | *Pteropus poliocephalus*^€,ↄ^ | | 1 | 0 |  | 0 |  | 0 |  | 0 |  | 0 | 1 | 0 |  | 0 |  | 0 |  | 0 |  | 0 |
|  | Suidae | Pig | *Sus scrofa** | | 1 | 0.09 |  | 0 |  | 0 |  | 0 | 1 | 0.07 | 1 | 0.09 |  | 0 | 1 | 0.28 |  | 0 | 1 | 1.08 |
|  | Tachyglossidae | Short-beaked echidna | *Tachyglossus aculeatus* | | 1 | 2.79 | 1 | 6.47 |  | 0 |  | 0 | 1 | 2.25 | 1 | 2.02 | 1 | 2.40 | 1 | 1.11 | 1 | 6.00 |  | 0 |
|  | Vespertilionidae | Lesser long-eared bat | *Nyctophilus geoffroyi* | | 1 | 0 |  | 0 |  | 0 |  | 0 |  | 0 | 1 | 0 |  | 0 |  | 0 |  | 0 |  | 0 |
|  |  | Undetermined long-eared bat | *Nyctophilus* sp. | | 1 | 0.03 |  | 0 |  | 0 |  | 0 | 1 | 0.04 | 1 | 0.09 |  | 0 |  | 0 |  | 0 |  | 0 |
|  |  | Undetermined microbat |  | | 1 | 0.02 |  | 0 |  | 0 |  | 0 | 1 | 0.04 |  | 0 |  | 0 |  | 0 |  | 0 |  | 0 |
|  | Vombatidae | Common wombat | *Vombatus ursinus* | | 1 | 11.86 |  | 0 |  | 0 | 1 | 4.55 | 1 | 15.14 | 1 | 11.43 | 1 | 12.02 | 1 | 8.33 | 1 | 29.50 |  | 0 |
| Fish |  | Undetermined fish | | | 1 | 0.03 |  | 0 |  | 0 |  | 0 |  | 0 |  | 0 |  | 0 | 1 | 0.28 |  | 0 | 1 | 1.08 |
| Bird |  | Undetermined bird | | | 1 | 4.17 | 1 | 10.74 | 1 | 8.57 | 1 | 4.55 | 1 | 2.62 | 1 | 1.93 | 1 | 2.64 | 1 | 9.17 | 1 | 3.50 | 1 | 6.45 |
|  | Dromaiidae | Emu | *Dromaius novaehollandiae* | | 1 | 0.02 |  | 0 | 1 | 2.86 |  | 0 |  | 0 |  | 0 |  | 0 |  | 0 |  | 0 |  | 0 |
|  | Menuridae | Lyrebird | *Menura novaehollandiae* | | 1 | 0 |  | 0 |  | 0 |  | 0 | 1 | 0 |  | 0 |  | 0 |  | 0 |  | 0 |  | 0 |
|  | Phasianidae | Fowl | *Gallus domesticus* | | 1 | 0 |  | 0 |  | 0 |  | 0 | 1 | 0 |  | 0 |  | 0 |  | 0 |  | 0 |  | 0 |
| Reptiles and amphibians | | Undetermined reptile or amphibian |  | | 1 | 0 |  | 0 |  | 0 |  | 0 |  | 0 | 1 | 0 |  | 0 |  | 0 |  | 0 |  | 0 |
| Table S1 (cont.) | | | | | | | | | | | | | | | | | | | | | | | | |
| Food item | Family (Order for insects) | Common name | Scientific name | | All regions | | Mallee | | Wimmera | | Northern Country | | North East | | East G | | West/South G | | Central | | North Central | | South West | |
|  |  |  |  | | P | % | P | % | P | % | P | % | P | % | P | % | P | % | P | % | P | % | P | % |
|  |  | Undetermined reptile | | | 1 | 1.34 | 1 | 10.62 |  | 0 |  | 0 | 1 | 0.26 | 1 | 0.97 | 1 | 0.48 |  | 0 |  | 0 |  | 0 |
|  |  | Undetermined snake | | | 1 | 0.09 | 1 | 0.12 | 1 | 2.86 |  | 0 | 1 | 0.07 |  | 0 |  | 0 | 1 | 0.28 |  | 0 |  | 0 |
|  |  | Undetermined skink | | | 1 | 0.02 | 1 | 0.12 |  | 0 |  | 0 |  | 0 |  | 0 |  | 0 |  | 0 |  | 0 |  | 0 |
|  | Varanidae | Undetermined goanna | *Varanus* sp. | | 1 | 0.02 |  | 0 |  | 0 |  | 0 |  | 0 | 1 | 0.09 |  | 0 |  | 0 |  | 0 |  | 0 |
|  | Agamidae | Undetermined dragon | *Pogona* sp.^ƹ^ | | 1 | 0.12 | 1 | 0.73 | 1 | 2.86 |  | 0 |  | 0 |  | 0 |  | 0 |  | 0 |  | 0 |  | 0 |
|  | Scincidae | Blue-tongued skink | *Tiliqua* sp.^ƹ^ | | 1 | 0.07 | 1 | 0.37 | 1 | 2.86 |  | 0 |  | 0 |  | 0 |  | 0 |  | 0 |  | 0 |  | 0 |
| Insects | Diptera and others | Undetermined insect |  | | 1 | 7.80 | 1 | 39.44 | 1 | 11.43 | 1 | 11.43 | 1 | 3.26 | 1 | 0.62 | 1 | 0.24 | 1 | 3.89 | 1 | 5.50 | 1 | 2.15 |
|  | Coleoptera or Blattodea | Undetermined beetle or cockroach | | | 1 | 0.31 | 1 | 1.47 | 1 | 14.29 |  | 0 | 1 | 0 |  | 0 |  | 0 | 1 | 0.28 |  | 0 |  | 0 |
|  | Hymenoptera or Blattodea | Undetermined ant or termite | | | 1 | 0.10 | 1 | 0.61 | 1 | 2.86 |  | 0 |  | 0 |  | 0 |  | 0 |  | 0 |  | 0 |  | 0 |
|  | Lepidoptera | Undetermined moth | *Antheruea eucalypti, Agrotis infusa* etc. | | 1 | 0.07 |  | 0 |  | 0 |  | 0 | 1 | 0.15 |  | 0 |  | 0 |  | 0 |  | 0 |  | 0 |
|  | Orthoptera | Undetermined grasshopper or cricket | | | 1 | 0.10 | 1 | 0.61 |  | 0 |  | 0 | 1 | 0.04 |  | 0 |  | 0 |  | 0 |  | 0 |  | 0 |
| Other invertebrates | | Undetermined crustacean | | | 1 | 0.02 |  | 0 |  | 0 |  | 0 |  | 0 | 1 | 0.09 |  | 0 |  | 0 |  | 0 |  | 0 |
|  | Parastacidae | Undetermined yabby | | | 1 | 0.05 |  | 0 |  | 0 |  | 0 |  | 0 |  | 0 |  | 0 | 1 | 0.28 | 1 | 1.00 |  | 0 |
|  |  | Undetermined mollusc | | | 1 | 0.02 |  | 0 |  | 0 |  | 0 |  | 0 |  | 0 |  | 0 |  | 0 | 1 | 0.50 |  | 0 |
| Plant material | | Undetermined plant material: | grass, leaves, twigs etc. | | 1 | 5.74 | 1 | 26.62 | 1 | 5.71 |  | 0 | 1 | 3.94 | 1 | 0.35 |  | 0 | 1 | 0.56 | 1 | 1.00 | 1 | 1.08 |
|  |  | Seeds |  | | 1 | 0.54 | 1 | 0.24 | 1 | 8.57 | 1 | 4.55 | 1 | 0.19 | 1 | 0.35 |  | 0 | 1 | 1.94 | 1 | 4.50 |  | 0 |
|  |  | Fruit |  | | 1 | 0.20 | 1 | 1.47 |  | 0 |  | 0 |  | 0 |  | 0 |  | 0 |  | 0 |  | 0 |  | 0 |
|  |  | Black berry fruit or seeds | *Rubus fruticosus* | | 1 | 0.10 |  | 0 |  | 0 |  | 0 | 1 | 0.04 |  | 0 |  | 0 | 1 | 1.39 |  | 0 |  | 0 |
| Unidentified material | | Bone, meat, fat, hide etc. | | | 1 | 1.40 | 1 | 4.88 |  | 0 | 1 | 6.82 | 1 | 0.41 | 1 | 0.53 | 1 | 0.24 | 1 | 3.33 |  | 0 | 1 | 4.30 |
| Eggshell | |  | | | 1 | 0.12 | 1 | 0.85 |  | 0 |  | 0 |  | 0 |  | 0 |  | 0 |  | 0 |  | 0 |  | 0 |
| Soil/detritis | |  | | | 1 | 0.07 |  | 0 | 1 | 2.86 |  | 0 | 1 | 0 |  | 0.26 |  | 0 |  | 0 |  | 0 |  | 0 |
| Rubbish | | Paper, plastic, fishing line | | | 1 | 0.12 |  | 0 |  | 0 |  | 0 | 1 | 0.04 |  | 0 |  | 0 | 1 | 1.67 |  | 0 |  | 0 |

S2 Table legend.

**Data are presence (P) records (‘1’ indicates present) based on all (raw and summary) data sources^1,2,3,4,5,6,7,8,9,10,11,12,13,14,15,16,17,18,19,20,21,22,23,24,25,26,27,28,29,30,31,32,33,34,35,36,37,38,39,a,b,c,d,e,f,g,h,i,j,k,l,m,n,o,p,q,r,s,t,u,v,w,x,y,z,aa,bb,cc,dd,ee,ff,gg,hh,ii,jj,kk,ll,mm,nn,oo^, and percentage frequency occurrence (%) based on raw data sources^11,15,16,30,36,a,b,c,d,e,f,g,h,i,j,k,l,m,n,o,p,q,r,s,t,u,v,w,x,y,z,aa,bb,cc,dd,ee,ff,gg,hh,ii,jj,kk,ll,mm,nn,oo^ across all regions of Victoria (raw data: *n* = 5875 scats; summary data^^: *n* = 2050^ scats, *n* = 805 stomachs, *n* = 19 scat/stomach) and within each region: Mallee (raw data: *n* = 819 scats); Wimmera (raw data: *n* = 35 scats); Northern Country (raw data: *n* = 44 scats); North East (raw data: *n* = 2668 scats; summary data: *n* = 490 scats, *n* = 101 stomachs, *n* = 19 scat/stomach); East Gippsland (G) (raw data: *n* = 1137 scats; summary data: *n* = 1530^ scats); West and South Gippsland (raw data: *n* = 416 scats; summary data: *n* = 78 scats); Central (raw data: *n* = 360 scats); North Central (raw data: *n* = 200 scats; summary data^^); and South West (raw data: *n* = 93 scats).

^excluding Peacock et al. 1992 for which only total sample size for dog and fox of 110 provided

^^no sample size provided

^ǂ^probable identification

*exotic species

^ƹ^species that have not previously been identified in the diet of dingoes/wild dogs in Victoria in published studies that have used scat or stomach analysis (note the agile antechinus may have previously been recorded as the brown antechinus)

^€^Threatened (FFG Act 1988)

^£^Endangered (EPBC Act 1999)

^≠^Vulnerable (EPBC Act 1999)

^∂^Critically Endangered (IUCN 2013)

^∏^Endangered (IUCN 2013)

^∫^Near Threatened (IUCN 2013)

^ↄ^Vulnerable (IUCN 2013)

^Ω^Least Concern (IUCN 2013)

**References**

1. Brown GW, Carr GW, Cherry KA, Craig SA, Horrocks GFB, et al. (1986) Flora and fauna of the Quadra forest block, East Gippsland, Victoria. Unpublished report. Melbourne: Department of Conservation, Forests and Lands.

2. Brown GW, Horrocks GFB, Lunt ID, Meggs RA, Sandiford EM (1987) Flora and fauna of the Noorinbee forest block, East Gippsland, Victoria. Unpublished report. Melbourne: Department of Conservation, Forests and Lands.

3. Brown GW, Horrocks GFB, Meggs RA, Opie AM, Westaway J (1988) Flora and fauna of proposed timber harvesting areas in the Grampians National Park, Victoria. Part II. Unpublished report. Melbourne: Department of Conservation, Forests and Lands.

4. Brown GW, Earl GE, Griffiths RC, Horrocks GFB, Williams LM (1989) Flora and fauna of the Acheron Forest Block, Central Highlands, Victoria. Unpublished report. Melbourne: Department of Conservation, Forests and Lands.

5. Brunner H (1978) The diet of dingoes and foxes in the Dartmouth Reservoir area. Victorian State Rivers and Water Supply Commission: Dartmouth Dam Project Report on Environmental Studies Part 2 pp. 19-28

6. Chesterfield EA, Macfarlane MA, Allen D, Hutchinson MN, Triggs B, et al. (1983) Flora and fauna of the Rodger Forest Block, East Gippsland, Victoria. Unpublished report. Forests Commission of Victoria.

7. Chesterfield EA, Hurley VA, Henry SR, Schulz M, Pyrke AF (1988) Flora and fauna of the Brodribb Forest block, East Gippsland, Victoria. Unpublished report. Melbourne: Department of Conservation, Forests and Lands.

8. Coman BJ (1972) Helminth parasites of the dingo and feral dog in Victoria with some notes on the diet of the host. Australian Veterinary Journal 48: 456-461.

9. Corbett LK (1974) Contributions to the biology of dingoes (Camivora: Canidae) in Victoria. Melbourne: Monash University. 197 p.

10. Davies JB, Carter RL, Drummond MB, Hollis GJ, Pascoe CG, et al. (1994) Flora and fauna of the Eastern and Western Tyers Forest Blocks and adjacent south-eastern slopes of Baw Baw National Park, central Gippsland, Victoria. Unpublished report. Department of Conservation and Natural Resources.

11. Earl GE, Brown GW, Cherry KA, Horrocks GFB, Vollebergh PJ (1989) Flora and fauna of the Ellery Forest Block (southern and eastern parts), East Gippsland, Victoria. Unpublished report. Melbourne: Department of Conservation, Forests and Lands.

12. Gillespie GR, Henry SR, Mueck SG, Scotts D, Westaway J (1990) Flora and fauna of the Pheasant Creek and Upper Buenba forest blocks, alpine area, Victoria. Ecological Survey Report No. 29, Department of Conservatoin and Environment, Victoria. Unpublished report.

13. Gillespie GR, Humphries R, Horrocks GFB, Lobert BO, McLaughlin J (1992) Flora and fauna of the Stony Peak and Genoa Forest Blocks, East Gippsland, Victoria. Unpublished report. Melbourne: Department of Conservation and Environment, Victoria.

14. Henry SR, Cherry KA, Hurley VA, Opie AM, Schulz M (1988) Flora and fauna of the Tennyson forest block, east Gippsland, Victoria. Unpublished report. Melbourne: Department of Conservation, Forests and Lands.

15. Hollis GJ, Carter RL, Davies JB, Drummond MB, Oates AM, et al. (1995) Survey of vegetation and vertebrate fauna in the Ada Forest Block, Port Phillip Area, Victoria. Unpublished report. Department of Conservation and Natural Resources.

16. Hollis GJ, Carter RL, Davies JB, Davies MJ, Drummond MB, et al. (1995) Survey of vegetation and vertebrate fauna in the Cascade Forest Block and adjacent areas of Baw Baw National Park, Central Gippsland, Victoria. Unpublished report. Department of Conservation and Natural Resources.

17. Horrocks GFB, Opie AM, Carr GW, Cherry KA, Brown GW, et al. (1984) Flora and fauna of the Ellery Forest Block, East Gippsland, Victoria. Unpublished report. Melbourne: Department of Conservation, Forests and Lands.

18. Lobert BO, Gillespie GR, Lunt ID, Peacock RJ, Robinson D (1991) Flora and fauna of the Goolengook Forest Block, East Gippsland, Victoria. Unpublished report. Melbourne: Department of Conservation and Environment, Victoria.

19. Loyn RH, Cameron DG, Traill BJ, Sloan JF, Malone BS, et al. (1992) Flora and fauna of the Cooaggalah Forest Block, East Gippsland, Victoria. Unpublished report. Melbourne: Department of Conservation and Environment, Victoria.

20. MacFarlane MA, Loyn RH, Chesterfield EA, Traill BJ, Triggs BE (1984) Flora and fauna of the Scorpion and Dawson Forest Blocks, East Gippsland, Victoria. Unpublished report. Melbourne: Department of Conservation, Forests and Lands.

21. Macfarlane MA, Schulz M, Parkes DM, Traill BJ, Triggs BE (1987) Flora and fauna of the Buckland forest block, east Gippsland, Victoria. Unpublished report. Melbourne: Department of Conservation, Forests and Lands.

22. Newsome AE, Catling PC, Corbett LK (1983) The feeding ecology of the dingo II. Dietary and numerical relationships with fluctuating prey populations in southeastern Australia. Australian Journal of Ecology 8 345–366.

23. Opie AM, Cherry KA, Horrocks GFB, Carr GW, Schulz M, et al. (1984) Flora and fauna of the Yalmy forest block, East Gippsland, Victoria. Unpublished report. Melbourne: Department of Conservation, Forests and Lands.

24. Opie AM, Brown GW, Carr GW, Cherry KA, Horrocks GFB, et al. (1987) Flora and fauna of the Splitters Range forest block, Bairnsdale Region, Victoria. Unpublished report. Department of Conservation, Forests and Lands.

25. Opie AM, Gillespie GR, Henry SR, Lobert BO, Pyrke AF (1990) Flora and fauna of the Coast Range Forest Block (southern part) East Gippsland, Victoria. Unpublished report. Melbourne: Department of Conservation and Environment.

26. Parkes DM, Macfarlane MA, Schulz M, Traill BJ (1987) Flora and fauna of the Nunniong North forest block, Bairnsdale region, Victoria. Unpublished report. Melbourne: Department of Conservation, Forests and Lands.

27. Peacock RJ, Brown GW, Duncan S, Gillespie GR, Robinson D, et al. (1992) Flora and fauna of the Sardine Forest Block, East Gippsland, Victoria. Unpublished report. Melbourne: Department of Conservation and Environment, Victoria.

28. Peel B, Bilney RJ, Bilney RJ (2005) Observations of the ecological impacts of Sambar *Cervus unicolor* in East Gippsland, Australia: with reference to destruction of rainforest communities. The Victorian Naturalist 122: 189-200.

29. Pyrke AF, Gillespie GR, Henry SR, Meggs RA, Westaway J (1988) Flora and fauna of the Clover and Pretty Valley Forest Blocks, north-east Victoria. Unpublished report. Melbourne: Department of Conservation, Forests and Lands.

30. Ritchie EG (2014) The impacts of wild dogs across the public-private land interface in North West Victoria. Unpublished report.

31. Robinson PD, Collins MG, Gillespie GR, Humphries R, Lobert BO, et al. (1992) Flora and fauna of the Saltpetre Forest Block, north-east Victoria. Unpublished report. Melbourne: Department of Conservation and Environment, Victoria.

32. Schulz M, Macfarlane MA, Parkes DM, Traill BJ, Triggs B, et al. (1987) Flora and fauna of the Mt Murray forest block, north-eastern Victoria. Unpublished report. Melbourne: Department of Conservation, Forests and Lands: Public Lands and Forest Division.

33. Stevens PL (1981) Wild dogs in Victoria. Department of Crown Lands and Survey, Victoria. Pamphlet no. 81.

34. Suckling GC, Backen E, Heislers A, Neuman FG (1976) The flora and fauna of radiata pine plantations in north-eastern Victoria. Forests Commission Victoria Bulletin 24: 1-58.

35. Triggs B, Brunner H, Cullen JM (1984) The food of fox, dog and cat in Croajingalong National Park, south-eastem Victoria. Australian Wildlife Research 11: 491-499.

36. Victoria College (1988) Forest regeneration, significant vegetation and small mammals in the Coast Range Forest Block, east Gippsland, Victoria. Unpublished reports. Faculty of Science, Victoria College, Melbourne.

37. Westaway J, Cherry KA, Duncan PE, Gillespie GR, Henry SR, et al. (1990) Flora and fauna of the Lower Wilkinson and Fainting Range forest blocks, Bairnsdale region, Victoria. Ecological Survey Report No. 27, Department of Conservation and Environment, Victoria. Unpublished report.

38. Westaway J, Henry SR, Gillespie GR, Lobert BO, Scotts DJ, et al. (1990) Flora and fauna of the west Errinundra and Delegate forest blocks, East Gippsland, Victoria. Unpublished report. Department of Conservation and Environment, Victoria.

39. Yugovic JV, Brown GW, Henry SR, Meggs RA (1987) Flora and fauna of the Sisters Forest Block, east Gippsland, Victoria. Unpublished report. Melbourne: Department of Conservation, Forests and Lands.

**Other data sources**

^a^Arthur Rylah Institue for Environmental Research, Department of Environment and Primary Industries (DEPI)

^b^Biosis

^c^DEPI

^d^DEPI Alexandra

^e^DEPI Benalla

^f^DEPI Corryong

^g^DEPI Hamilton

^h^DEPI Horsham

^i^DEPI Mallacoota

^j^DEPI Mildura

^k^DEPI Orbost

^l^DEPI Portland

^m^DEPI Tallangatta

^n^DEPI Wangaratta

^o^DEPI Warragul

^p^DEPI Wodonga

^q^Ecology Australia

^r^Gippsland High Country Tours

^s^Melbourne Water

^t^Mt Buller & Mt Stirling Alpine Resort Management Board

^u^Parks Victoria (PV)

^v^PV Braeside

^w^PV Cann River

^x^PV Dargo

^y^PV Dimboola

^z^PV Foster

^aa^PV Gembrook

^bb^PV Halls Gap

^cc^PV Heyfield

^dd^PV Lysterfield-Berwick

^ee^PV Mallacoota

^ff^PV Mansfield

^gg^PV Mildura

^hh^PV Sale

^ii^PV Tallangatta

^jj^PV Wangaratta

^kk^PV Wilsons Promontory National Park

^ll^Victoria Malleefowl Recovery Group

^mm^Wild Ecology

^nn^Wildlife Unlimited

^oo^Winton Wetlands Committee of Management
