# Supplementary material for: Interspecific and Geographic Variation in the Diets of Sympatric Carnivores: Dingoes/Wild Dogs and Red Foxes in South-Eastern Australia
Source: PLoS One. 2015 Mar 19;10(3):e0120975. doi: 10.1371/journal.pone.0120975 (PMC4366095; doi:10.1371/journal.pone.0120975)
Supplement: S2 Table — (DOCX) [file pone.0120975.s003.docx]

**S3 Table.** Checklist of taxa recorded in the diet of foxes in Victoria.**

| Food item | Family (Order for insects) | Common name | Scientific name | All regions | | Mallee | | Wimmera | | Northern Country | | North East | | East G | | West/South G | | Central | | North Central | | South West | |
| --- | --- | --- | --- | --- | --- | --- | --- | --- | --- | --- | --- | --- | --- | --- | --- | --- | --- | --- | --- | --- | --- | --- | --- |
|  |  |  |  | P | % | P | % | P | % | P | % | P | % | P | % | P | % | P | % | P | % | P | % |
| Mammal | Acrobatidae | Feathertail glider | *Acrobates pygmaeus* | 1 | 0.09 |  | 0 |  | 0 |  | 0 | 1 | 0.14 | 1 | 0.28 | 1 | 0.44 | 1 | 0 |  | 0 |  | 0 |
|  | Bovidae | Sheep | *Ovis aries** | 1 | 7.23 | 1 | 12.89 | 1 | 11.64 | 1 | 16.33 | 1 | 0.78 | 1 | 0.38 | 1 | 0.22 | 1 | 4.45 |  | 0 | 1 | 2.27 |
|  |  | Cattle | *Bos taurus** | 1 | 0.70 | 1 | 0.89 | 1 | 0.53 | 1 | 2.04 | 1 | 0.34 | 1 | 0.75 | 1 | 0.44 | 1 | 1.27 |  | 0 | 1 | 0 |
|  | Burramyidae | Mountain pygmy-possum | *Burramys parvus*^€,£,∂,ƹ^ | 1 | 0.18 |  | 0 |  | 0 |  | 0 | 1 | 0.72 |  | 0 |  | 0 |  | 0 |  | 0 |  | 0 |
|  |  | Eastern pygmy-possum | *Cercartetus nanus* | 1 | 0.54 |  | 0 |  | 0 |  | 0 | 1 | 1.09 | 1 | 2.35 | 1 | 0.65 | 1 | 0.16 |  | 0 | 1 | 0.32 |
|  |  | Little pygmy-possum | *Cercartetus lepidus*^ƹ^ | 1 | 0.44 | 1 | 0.91 |  | 0 |  | 0 |  | 0 |  | 0 |  | 0 |  | 0 |  | 0 |  | 0 |
|  |  | Undeteremined pygmy-possum | *Cercartetus* sp. | 1 | 0.03 | 1 | 0.05 |  | 0 |  | 0 |  | 0 |  | 0 |  | 0 |  | 0 |  | 0 |  | 0 |
|  | Canidae | Dingo/dog | *Canis dingo/familiaris** | 1 | 0.10 | 1 | 0.09 |  | 0 | 1 | 1.02 |  | 0 | 1 | 0.09 |  | 0 | 1 | 0.32 |  | 0 | 1 | 0.65 |
|  |  | Red fox | *Vulpes vulpes** | 1 | 0.11 | 1 | 0.07 | 1 | 1.06 |  | 0 | 1 | 0.17 | 1 | 0 |  | 0 | 1 | 0.16 |  | 0 | 1 | 0 |
|  | Caprinae | Goat | *Capra hircu*s* | 1 | 0.59 | 1 | 1.00 | 1 | 1.59 |  | 0 | 1 | 0.14 | 1 | 0 | 1^ǂ^ | 0.22 | 1 | 0.32 |  | 0 | 1 | 0.32 |
|  | Cervidae | Sambar deer | *Rusa unicolor**^,ƹ^ | 1 | 0.24 |  | 0 |  | 0 |  | 0 | 1 | 0.48 |  | 0 | 1 | 2.61 |  | 0 |  | 0 |  | 0 |
|  |  | Red deer | *Cervus elaphus scoticus**^,ƹ^ | 1 | 0.02 |  | 0 | 1 | 1.06 |  | 0 |  | 0 |  | 0 |  | 0 |  | 0 |  | 0 |  | 0 |
|  |  | Hog deer | *Axis porcinus**^,ƹ^ | 1 | 0.04 |  | 0 |  | 0 |  | 0 |  | 0 |  | 0 | 1 | 1.09 |  | 0 |  | 0 |  | 0 |
|  |  | Undetermined deer* |  | 1 | 0.03 |  | 0 | 1 | 0.53 |  | 0 | 1 | 0.03 |  | 0 | 1 | 0.22 | 1 | 0.16 |  | 0 |  | 0 |
|  | Dasyuridae | Brown antechinus | *Antechinus stuartii* | 1 | 1.18 |  | 0 |  | 0 |  | 0 | 1 | 2.39 | 1 | 5.08 | 1 | 1.53 | 1 | 0.16 |  | 0 | 1 | 1.30 |
|  |  | Dusky antechinus | *Antechinus swainsonii* | 1 | 4.30 |  | 0 |  | 0 |  | 0 | 1 | 9.21 | 1 | 8.60 | 1 | 7.19 | 1 | 1.75 | 1 | 56.35 | 1 | 2.27 |
|  |  | Swamp antechinus | *Antechinus minimus*^Ω^ | 1 | 0.03 |  | 0 |  | 0 |  | 0 |  | 0 | 1 | 0 | 1 | 0.44 |  | 0 |  | 0 | 1 | 0.32 |
|  |  | Agile antechinus | *Antechinus agilis* | 1 | 0.29 |  | 0 | 1 | 1.59 |  | 0 | 1 | 0.31 | 1 | 1.41 | 1 | 0.44 | 1 | 0.79 |  | 0 |  | 0 |
|  |  | Yellow-footed antechinus | *Antechinus flavipes*^ƹ^ | 1 | 0.09 |  | 0 |  | 0 |  | 0 | 1 | 0 |  | 0 |  | 0 |  | 0 |  | 0 | 1 | 2.92 |
|  |  | Undetermined antichinus | *Antechinus* sp. | 1 | 0.31 |  | 0 | 1^ǂ^ | 0.53 |  | 0 | 1 | 0.41 | 1 | 1.03 | 1 | 0.65 | 1 | 0.48 |  | 0 | 1 | 1.30 |
|  |  | Mallee ningaui | *Ningaui yvonneae*^ƹ^ | 1 | 0.50 | 1 | 1.04 |  | 0 |  | 0 |  | 0 |  | 0 |  | 0 |  | 0 |  | 0 |  | 0 |
| Table S2 (cont.) | | | | | | | | | | | | | | | | | | | | | | | |
| Food item | Family (Order for insects) | Common name | Scientific name | All regions | | Mallee | | Wimmera | | Northern Country | | North East | | East G | | West/South G | | Central | | North Central | | South West | |
|  |  |  |  | P | % | P | % | P | % | P | % | P | % | P | % | P | % | P | % | P | % | P | % |
|  |  | White-footed dunnart | *Sminthopsis leucopus*^€,≠^ | 1 | 0.03 |  | 0 |  | 0 |  | 0 | 1 | 0 | 1 | 0.28 |  | 0 |  | 0 |  | 0 |  | 0 |
|  |  | Common dunnart | *Sminthopsis murina* | 1 | 0.19 | 1 | 0.38 |  | 0 | 1 | 1.02 | 1 | 0 |  | 0 |  | 0 |  | 0 |  | 0 |  | 0 |
|  |  | Fat-tailed dunnart | *Sminthopsis crassicaudata*^ƹ^ | 1 | 0.08 | 1 | 0.16 |  | 0 |  | 0 |  | 0 |  | 0 |  | 0 |  | 0 |  | 0 |  | 0 |
|  |  | Undetermined dunnart | *Sminthopsis* sp. | 1 | 0.16 | 1 | 0.32 |  | 0 |  | 0 | 1 | 0 |  | 0 |  | 0 |  | 0 |  | 0 |  | 0 |
|  |  | Undetermined dasyurid |  | 1 | 0.02 | 1 | 0.02 | 1 | 0.53 |  | 0 |  | 0 |  | 0 |  | 0 |  | 0 |  | 0 |  | 0 |
|  | Felidae | Cat | *Felis catus** | 1 | 0.16 | 1 | 0.13 | 1 | 0.53 | 1 | 1.02 | 1 | 0.27 |  | 0 |  | 0 | 1 | 0.16 |  | 0 | 1 | 0.32 |
|  | Hominidae | Human | *Homo sapiens** | 1 | 0.03 |  | 0 |  | 0 |  | 0 | 1 | 0.07 |  | 0 |  | 0 | 1 | 0.32 |  | 0 |  | 0 |
|  | Leporidae | European rabbit | *Oryctolagus cuniculus** | 1 | 16.86 | 1 | 18.22 | 1 | 11.64 | 1 | 23.47 | 1 | 17.40 | 1 | 8.46 | 1 | 11.76 | 1 | 30.05 | 1 | 3.31 | 1 | 2.27 |
|  |  | Brown hare | *Lepus capensis** | 1 | 0.48 | 1 | 0.04 | 1 | 1.06 |  | 0 | 1 | 0.89 | 1^ǂ^ | 0.38 | 1^ǂ^ | 0.44 | 1 | 2.38 | 1^ǂ^ | 1.66 |  | 0 |
|  |  | Undetermined lagomorph* |  | 1 | 0.43 |  | 0 | 1 | 0.53 |  | 0 | 1 | 0.55 | 1 | 0.38 | 1 | 2.40 | 1 | 2.23 | 1 | 1.66 |  | 0 |
|  | Macropodidae | Red-necked wallaby | *Macropus rufogriseus* | 1 | 0.04 | 1 | 0.02 |  | 0 |  | 0 |  | 0 | 1 | 0.28 |  | 0 |  | 0 |  | 0 | 1 | 0.32 |
|  |  | Black wallaby | *Wallabia bicolor* | 1 | 4.17 | 1 | 0.04 | 1 | 15.87 | 1 | 6.12 | 1 | 6.89 | 1 | 10.53 | 1 | 15.69 | 1 | 3.50 |  | 0 | 1 | 7.79 |
|  |  | Black wallaby or Red-necked wallaby | *W. bicolor* or *M. rufogriseus* | 1 | 0.01 |  | 0 |  | 0 |  | 0 |  | 0 | 1 | 0.09 |  | 0 | 1 | 0 |  | 0 |  | 0 |
|  |  | Eastern grey kangaroo | *Macropus giganteus* | 1 | 0.38 |  | 0 | 1 | 2.65 | 1 | 7.14 | 1 | 0.75 | 1 | 0.38 | 1 | 0.22 | 1 | 0.48 |  | 0 | 1 | 0.65 |
|  |  | Western grey kangaroo | *Macropus fuliginosus*^ƹ^ | 1 | 3.21 | 1 | 5.81 | 1 | 5.82 |  | 0 |  | 0 |  | 0 |  | 0 |  | 0 |  | 0 | 1 | 11.04 |
|  |  | Red kangaroo | *Macropus rufus*^ƹ^ | 1 | 0.83 | 1 | 1.72 |  | 0 |  | 0 |  | 0 |  | 0 |  | 0 |  | 0 |  | 0 |  | 0 |
|  |  | Eastern wallaroo | *Macropus robustus robustus*^€,Ω,ƹ^ | 1 | 0.03 |  | 0 |  | 0 |  | 0 | 1 | 0.10 |  | 0 |  | 0 |  | 0 |  | 0 |  | 0 |
|  |  | Undetermined kangaroo or wallaby | *Macropus* sp. | 1 | 0.90 | 1 | 1.72 | 1 | 2.65 |  | 0 | 1 | 0.03 | 1 | 0.09 |  | 0 |  | 0 |  | 0 | 1 | 0.32 |
|  |  | Undetermined macropod | | 1 | 0.50 | 1 | 0.68 | 1 | 3.17 | 1 | 1.02 | 1 | 0.10 | 1 | 0.19 | 1 | 0.44 | 1 | 0.32 |  | 0 | 1 | 0.65 |
|  | Muridae | Water rat | *Hydromys chrysogaster* | 1 | 0.18 |  | 0 |  | 0 |  | 0 | 1 | 0.24 | 1 | 0.56 | 1 | 0.44 | 1 | 0.32 | 1 | 2.21 |  | 0 |
|  |  | Broad-toothed rat | *Mastacomys fuscus*^€,∫^ | 1 | 1.52 |  | 0 |  | 0 |  | 0 | 1 | 4.95 | 1 | 0.47 | 1 | 0.44 | 1 | 0.16 | 1 | 12.71 | 1 | 0 |
|  |  | House mouse | *Mus musculus** | 1 | 9.27 | 1 | 17.35 | 1 | 0.53 | 1 | 8.16 | 1 | 1.13 | 1 | 1.03 | 1 | 2.40 | 1 | 5.09 |  | 0 | 1 | 1.30 |
| Table S2 (cont.) | | | | | | | | | | | | | | | | | | | | | | | |
| Food item | Family (Order for insects) | Common name | Scientific name | All regions | | Mallee | | Wimmera | | Northern Country | | North East | | East G | | West/South G | | Central | | North Central | | South West | |
|  |  |  |  | P | % | P | % | P | % | P | % | P | % | P | % | P | % | P | % | P | % | P | % |
|  |  | Mitchell's hopping-mouse | *Notomys mitchellii*^ƹ^ | 1 | 1.34 | 1 | 2.77 |  | 0 |  | 0 |  | 0 |  | 0 |  | 0 |  | 0 |  | 0 |  | 0 |
|  |  | Smoky mouse | *Pseudomys fumeus*^€,£,∏,ƹ^ | 1 | 0.08 |  | 0 | 1 | 1.06 |  | 0 | 1 | 0.20 |  | 0 | 1 | 0.22 |  | 0 |  | 0 |  | 0 |
|  |  | Silky mouse | *Pseudomys apodemoides*^ƹ^ | 1 | 0.12 | 1 | 0.25 |  | 0 |  | 0 |  | 0 |  | 0 |  | 0 |  | 0 |  | 0 |  | 0 |
|  |  | Heath mouse | *Pseudomys shortridgei*^€,∫,ƹ^ | 1 | 0.01 |  | 0 | 1 | 0.53 |  | 0 |  | 0 |  | 0 |  | 0 |  | 0 |  | 0 |  | 0 |
|  |  | Bush rat | *Rattus fuscipes* | 1 | 7.22 |  | 0 |  | 0 |  | 0 | 1 | 22.74 | 1 | 13.35 | 1 | 8.71 | 1 | 0.95 | 1 | 19.34 | 1 | 0.65 |
|  |  | Swamp rat | *Rattus lutreolus* | 1 | 0.89 |  | 0 | 1 | 0.53 |  | 0 | 1 | 0.07 | 1 | 4.23 | 1 | 3.49 | 1 | 1.91 |  | 0 | 1 | 7.79 |
|  |  | Brown rat | *Rattus norvegicus* | 1 | 0.03 |  | 0 |  | 0 |  | 0 |  | 0 |  | 0 |  | 0 | 1 | 0.64 |  | 0 |  | 0 |
|  |  | Black rat | *Rattus rattus* | 1 | 0.78 | 1 | 0.05 |  | 0 |  | 0 | 1 | 1.09 | 1 | 1.13 | 1 | 1.53 | 1 | 4.77 |  | 0 | 1^ǂ^ | 1.62 |
|  |  | Undetermined rat | *Rattus* sp. | 1 | 1.45 |  | 0 | 1 | 1.06 | 1^ǂ^ | 1.02 | 1 | 2.66 | 1 | 3.85 | 1 | 3.49 | 1 | 2.86 |  | 0 | 1 | 3.25 |
|  |  | Undetermined rat | *Rattus* sp. or *Mastacomys* sp. | 1 | 0.01 |  | 0 |  | 0 |  | 0 |  | 0 | 1 | 0 |  | 0 | 1 | 0.16 |  | 0 |  | 0 |
|  |  | Undetermined rodent |  | 1 | 0.56 | 1 | 0.32 | 1 | 1.59 | 1 | 1.02 | 1 | 0.89 | 1 | 0.09 | 1 | 1.53 | 1 | 0.16 | 1 | 1.10 | 1 | 1.30 |
|  | Ornithorhynchidae | Platypus | *Ornithorhynchus anatinus*^ƹ^ | 1 | 0.12 |  | 0 |  | 0 |  | 0 | 1 | 0.14 | 1 | 0.56 | 1 | 0.87 |  | 0 |  | 0 |  | 0 |
|  | Otariidae | Australian fur seal | *Arctocephalus pusillus doriferus*^ƹ^ | 1 | 0.02 |  | 0 |  | 0 |  | 0 |  | 0 |  | 0 | 1 | 0.44 |  | 0 |  | 0 |  | 0 |
|  | Peramelidae | Long-nosed bandicoot | *Peremeles nasuta* | 1 | 1.75 |  | 0 |  | 0 |  | 0 | 1 | 3.21 | 1 | 8.55 | 1 | 3.27 | 1 | 0.48 |  | 0 | 1 | 0 |
|  |  | Southern brown bandicoot | *Isoodon obesulus obesulus*^€,£,Ω^ | 1 | 0.15 |  | 0 |  | 0 |  | 0 | 1 | 0 | 1 | 0.75 |  | 0 | 1 | 0 |  | 0 | 1 | 2.60 |
|  |  | Undetermined bandicoot |  | 1 | 0.03 |  | 0 |  | 0 |  | 0 |  | 0 | 1 | 0.19 |  | 0 | 1 | 0.16 |  | 0 |  | 0 |
|  | Petauridae | Yellow-bellied glider | *Petaurus australis* | 1 | 0.22 |  | 0 |  | 0 |  | 0 | 1 | 0.75 | 1 | 0.38 |  | 0 |  | 0 |  | 0 |  | 0 |
|  |  | Sugar glider | *Petaurus breviceps* | 1 | 0.63 |  | 0 | 1^ǂ^ | 1.06 | 1^ǂ^ | 7.14 | 1 | 1.06 | 1 | 2.44 | 1 | 0.65 | 1 | 0.48 |  | 0 |  | 0 |
|  |  | Squirrel glider | *Petaurus norfolcensis*^€,Ω,ƹ^ | 1^ǂ^ | 0.12 |  | 0 | 1^ǂ^ | 1.06 | 1^ǂ^ | 7.14 | 1^ǂ^ | 0.14 |  | 0 |  | 0 |  | 0 |  | 0 |  | 0 |
|  |  | Leadbeater's possum | *Gymnobelideus leadbeateri*^€,£,∏^ | 1 | 0 |  | 0 |  | 0 |  | 0 | 1 | 0 |  | 0 |  | 0 |  | 0 |  | 0 |  | 0 |
|  |  | Undetermined glider | *Petaurus* sp. | 1 | 0.16 |  | 0 | 1 | 1.06 | 1 | 7.14 | 1 | 0.31 |  | 0 |  | 0 |  | 0 |  | 0 |  | 0 |
| Table S2 (cont.) | | | | | | | | | | | | | | | | | | | | | | | |
| Food item | Family (Order for insects) | Common name | Scientific name | All regions | | Mallee | | Wimmera | | Northern Country | | North East | | East G | | West/South G | | Central | | North Central | | South West | |
|  |  |  |  | P | % | P | % | P | % | P | % | P | % | P | % | P | % | P | % | P | % | P | % |
|  | Phascolarctidae | Koala | *Phascolarctos cinereus* | 1 | 0.02 |  | 0 |  | 0 |  | 0 |  | 0 |  | 0 |  | 0 | 1 | 0.32 |  | 0 |  | 0 |
|  | Phalangeridae | Common brushtail possum | *Trichosurus vulpecula* | 1 | 1.30 | 1 | 0.39 | 1 | 2.12 |  | 0 | 1 | 1.50 | 1 | 0.56 | 1 | 3.92 | 1 | 5.25 |  | 0 | 1 | 6.82 |
|  |  | Mountain brushtail possum | *Trichosurus caninus* | 1 | 0.48 |  | 0 |  | 0 |  | 0 | 1 | 1.64 | 1 | 0.56 | 1^ǂ^ | 0.22 | 1 | 0.32 |  | 0 |  | 0 |
|  |  | Undetermined brushtail possum | *Trichosurus* sp. | 1 | 6.11 | 1 | 0.54 | 1 | 10.58 | 1 | 8.16 | 1 | 14.94 | 1 | 14.38 | 1 | 12.85 | 1 | 11.29 | 1 | 2.21 | 1 | 8.44 |
|  | Potoroidae | Long-footed potoroo | *Potorous longipes*^€,£,∏^ | 1 | 0.10 |  | 0 |  | 0 |  | 0 | 1 | 0.27 | 1 | 0.19 |  | 0 |  | 0 |  | 0 |  | 0 |
|  |  | Long-nosed potoroo | *Potorous tridactylus tridactylus*^€,≠,Ω^ | 1 | 0.16 |  | 0 |  | 0 |  | 0 | 1 | 0.03 | 1 | 1.03 | 1 | 0.22 | 1 | 0.16 |  | 0 | 1 | 1.30 |
|  |  | Undetermined potoroo | *Potorous* sp. | 1 | 0.01 |  | 0 |  | 0 |  | 0 |  | 0 | 1 | 0.09 |  | 0 |  | 0 |  | 0 |  | 0 |
|  | Pseudocheiridae | Common ringtail possum | *Pseudocheirus peregrinus* | 1 | 4.72 |  | 0 | 1 | 5.82 | 1 | 4.08 | 1 | 4.30 | 1 | 12.83 | 1 | 18.08 | 1 | 13.83 | 1 | 16.02 | 1 | 19.81 |
|  |  | Greater glider | *Petauroides volans* | 1 | 0.65 |  | 0 |  | 0 |  | 0 | 1 | 1.98 | 1 | 0.75 | 1 | 1.09 | 1 | 0.48 |  | 0 |  | 0 |
|  | Pseudocheiridae or Petauridae | Unidentified possum/glider | *Petauroides* sp. or *Petaurus* sp. | 1 | 0.03 |  | 0 |  | 0 |  | 0 | 1 | 0.07 | 1 | 0.19 |  | 0 |  | 0 |  | 0 |  | 0 |
|  | Phalangeridae, Pseudocheiridae or Peauridae | Unidentified possum/glider | *Trichosurus* sp., *P. peregrinus*, *Petaurus* sp. or *Petauroides* sp. | 1 | 0.06 |  | 0 |  | 0 |  | 0 | 1 | 0 | 1 | 0.28 | 1 | 0.65 | 1 | 0 |  | 0 | 1 | 0 |
|  | Pteropodidae | Grey-headed flying-fox | *Pteropus poliocephalus*^€,ↄ^ | 1 | 0.01 |  | 0 |  | 0 |  | 0 |  | 0 | 1 | 0.09 |  | 0 |  | 0 |  | 0 |  | 0 |
|  | Suidae | Pig | *Sus scrofa** | 1 | 0.02 |  | 0 |  | 0 |  | 0 | 1 | 0.03 | 1 | 0 |  | 0 | 1 | 0.16 |  | 0 |  | 0 |
|  | Tachyglossidae | Short-beaked echidna | *Tachyglossus aculeatus* | 1 | 0.33 | 1 | 0.38 |  | 0 |  | 0 | 1 | 0.20 | 1 | 0.38 | 1 | 0.86 | 1 | 0.16 |  | 0 | 1 | 0.32 |
|  | Vespertilionidae | Lesser long-eared bat | *Nyctophilus geoffroyi* | 1 | 0.01 |  | 0 |  | 0 |  | 0 | 1^ǂ^ | 0.03 |  | 0 |  | 0 |  | 0 |  | 0 | 1 | 0 |
|  |  | Undetermined long-eared bat | *Nyctophilus* sp. | 1^ǂ^ | 0.02 |  | 0 |  | 0 |  | 0 |  | 0 |  | 0 | 1^ǂ^ | 0.44 |  | 0 |  | 0 |  | 0 |
|  |  | Undetermined wattled bat | *Chalinolobus* sp.^ƹ^ | 1^ǂ^ | 0.01 |  | 0 |  | 0 |  | 0 |  | 0 | 1^ǂ^ | 0.09 |  | 0 |  | 0 |  | 0 |  | 0 |
|  |  | Undetermined microbat |  | 1 | 0.04 | 1 | 0.02 |  | 0 |  | 0 | 1 | 0.03 | 1 | 0.09 | 1 | 0.44 |  | 0 |  | 0 |  | 0 |
| Table S2 (cont.) | | | | | | | | | | | | | | | | | | | | | | | |
| Food item | Family (Order for insects) | Common name | Scientific name | All regions | | Mallee | | Wimmera | | Northern Country | | North East | | East G | | West/South G | | Central | | North Central | | South West | |
|  |  |  |  | P | % | P | % | P | % | P | % | P | % | P | % | P | % | P | % | P | % | P | % |
|  | Vombatidae | Common wombat | *Vombatus ursinus* | 1 | 0.77 |  | 0 |  | 0 |  | 0 | 1 | 1.57 | 1 | 1.03 | 1 | 5.01 | 1 | 0.48 | 1 | 2.76 |  | 0 |
| Bird |  | Undetermined bird |  | 1 | 7.27 | 1 | 9.26 | 1 | 7.94 | 1 | 9.18 | 1 | 3.65 | 1 | 4.89 | 1 | 8.93 | 1 | 11.76 |  | 0 | 1 | 4.87 |
|  | Charadriidae | Masked lapwing | *Vanellus miles* | 1 | 0 |  | 0 |  | 0 |  | 0 |  | 0 |  | 0 |  | 0 | 1 | 0 |  | 0 |  | 0 |
|  | Cracticidae | Australian magpie | *Gymnorhina tibicen* | 1 | 0 |  | 0 |  | 0 |  | 0 |  | 0 |  | 0 |  | 0 | 1 | 0 |  | 0 |  | 0 |
|  | Phasianidae | Fowl | *Gallus domesticus* | 1 | 0 |  | 0 |  | 0 |  | 0 | 1 | 0 |  | 0 |  | 0 |  | 0 |  | 0 |  | 0 |
|  | Procellariidae | Short tailed shearwater | *Puffinus tenuirostris* | 1 | 0 |  | 0 |  | 0 |  | 0 |  | 0 |  | 0 | 1 | 0 | 1 | 0 |  | 0 |  | 0 |
|  | Spheniscidae | Little penguin | *Eudyptula minor* | 1 | 0 |  | 0 |  | 0 |  | 0 |  | 0 |  | 0 |  | 0 | 1 | 0 |  | 0 |  | 0 |
| Reptiles and amphibians | | Undetermined reptile or amphibian |  | 1 | 0.24 |  | 0 |  | 0 | 1 | 0 | 1 | 0.10 | 1 | 0 | 1 | 0 | 1 | 0.16 |  | 0 | 1 | 0 |
|  |  | Undetermined reptile |  | 1 | 7.61 | 1 | 15.43 | 1 | 1.06 |  | 0 | 1 | 0.51 | 1 | 1.41 | 1 | 0.22 | 1 | 0.16 |  | 0 | 1 | 1.62 |
|  |  | Undetermined snake |  | 1 | 0.08 | 1 | 0.07 |  | 0 |  | 0 |  | 0 | 1 | 0.38 |  | 0 |  | 0 |  | 0 |  | 0 |
|  | Scincidae | Blue-tongued skink | *Tiliqua* sp.^ƹ^ | 1 | 0.01 |  | 0 |  | 0 |  | 0 |  | 0 |  | 0 | 1 | 0.22 |  | 0 |  | 0 |  | 0 |
| Insects |  | Undetermined insect |  | 1 | 37.11 | 1 | 67.79 | 1 | 28.57 | 1 | 16.33 | 1 | 8.05 | 1 | 2.26 | 1 | 7.41 | 1 | 8.59 | 1 | 0.55 | 1 | 16.56 |
|  | Lepidoptera | Undetermined moth |  | 1 | 1.46 |  | 0 |  | 0 |  | 0 | 1 | 5.46 |  | 0 |  | 0 |  | 0 |  | 0 | 1 | 2.92 |
|  |  | Undetermined caterpilla |  | 1 | 0.02 |  | 0 |  | 0 |  | 0 | 1 | 0.03 |  | 0 |  | 0 | 1 | 0.16 |  | 0 |  | 0 |
|  | Orthoptera | Undetermined grasshopper or cricket |  | 1 | 0 |  | 0 |  | 0 |  | 0 | 1 | 0 |  | 0 |  | 0 |  | 0 |  | 0 |  | 0 |
|  | Gryllidae | Black cricket | *Gryllus servillei* | 1 | 0 |  | 0 |  | 0 |  | 0 |  | 0 |  | 0 |  | 0 |  | 0 |  | 0 | 1 | 0 |
|  | Acrididae | Plague cricket | *Chortoicetes terminifera* | 1 | 0 |  | 0 |  | 0 |  | 0 |  | 0 |  | 0 |  | 0 |  | 0 |  | 0 | 1 | 0 |
|  | Gryllotalpidae | Mole cricket | *Gryllotalpa australia* | 1 | 0 |  | 0 |  | 0 |  | 0 |  | 0 |  | 0 |  | 0 |  | 0 |  | 0 | 1 | 0 |
|  | Coleoptera or Blattodea | Undetermined beetle or cockroach |  | 1 | 0.01 |  | 0 |  | 0 |  | 0 | 1 | 0 |  | 0 |  | 0 | 1 | 0.16 |  | 0 | 1 | 0 |
|  | Hemiptera: Cicadidae | Undetermined cicada | *Magicicada* sp. | 1 | 0 |  | 0 |  | 0 |  | 0 | 1 | 0 |  | 0 |  | 0 |  | 0 |  | 0 |  | 0 |
|  |  |  |  |  |  |  |  |  |  |  |  |  |  |  |  |  |  |  |  |  |  |  |  |
| Table S2 (cont.) | | | | | | | | | | | | | | | | | | | | | | | |
| Food item | Family (Order for insects) | Common name | Scientific name | All regions | | Mallee | | Wimmera | | Northern Country | | North East | | East G | | West/South G | | Central | | North Central | | South West | |
|  |  |  |  | P | % | P | % | P | % | P | % | P | % | P | % | P | % | P | % | P | % | P | % |
|  | Odonata | Undetermined dragonfly or damselfly |  | 1 | 0 |  | 0 |  | 0 |  | 0 | 1 | 0 |  | 0 |  | 0 |  | 0 |  | 0 |  | 0 |
| Other invertebrates | Mictyridae | Soldier crab | *Mictyrus platycheles* | 1 | 0 |  | 0 |  | 0 |  | 0 |  | 0 |  | 0 | 1 | 0 |  | 0 |  | 0 |  | 0 |
|  |  | Undetermined crustacean | *Engaeus victoriensis^∫^* etc. | 1 | 0.12 | 1 | 0.02 |  | 0 | 1 | 1.02 | 1 | 0.07 | 1 | 0.38 | 1 | 0.44 | 1 | 0.64 |  | 0 | 1 | 0 |
|  |  | Undetermined annelid |  | 1 | 0 |  | 0 |  | 0 |  | 0 | 1 | 0 |  | 0 |  | 0 |  | 0 |  | 0 | 1 | 0 |
|  |  | Undetermined mollusc |  | 1 | 0.01 |  | 0 |  | 0 |  | 0 |  | 0 |  | 0 |  | 0 | 1 | 0 |  | 0 |  | 0 |
|  |  | Undetermined arthropod |  | 1 | 0 |  | 0 |  | 0 |  | 0 | 1 | 0 |  | 0 |  | 0 |  | 0 |  | 0 | 1 | 0 |
|  |  | Undetermined invertebrate |  | 1 | 0 |  | 0 |  | 0 | 1 | 0 |  | 0 | 1 | 0 | 1 | 0.22 | 1 | 0 |  | 0 | 1 | 0 |
| Plant material | | Undetermined plant material: | grass, leaves, twigs etc. | 1 | 11.37 | 1 | 20.53 | 1 | 4.23 | 1 | 2.04 | 1 | 2.12 | 1 | 0.28 | 1 | 2.61 | 1 | 5.41 |  | 0 | 1 | 13.96 |
|  |  | Seeds: plums, apples, pears etc. | | 1 | 0.99 |  | 0 | 1 | 1.59 | 1 | 2.04 | 1 | 1.88 | 1 | 2.63 | 1 | 0.22 | 1 | 3.34 |  | 0 | 1 | 0 |
|  |  | Fruit |  | 1 | 0.17 | 1 | 0.21 |  | 0 |  | 0 | 1 | 0.27 |  | 0 |  | 0 | 1 | 0 |  | 0 |  | 0 |
|  |  | Black berry fruit or seeds | *Rubus fruticosus* | 1 | 0.22 |  | 0 |  | 0 |  | 0 | 1 | 0.38 | 1 | 0.09 | 1 | 0.87 | 1 | 1.43 |  | 0 | 1 | 0 |
| Unidentified material | | Bone, meat, fat, hide etc. |  | 1 | 0.22 | 1 | 0.14 |  | 0 | 1 | 0 | 1 | 0.17 | 1 | 0 | 1 | 0.22 | 1 | 0.32 |  | 0 | 1 | 2.92 |
| Eggshell |  |  |  | 1 | 0.81 | 1 | 1.66 |  | 0 |  | 0 |  | 0 |  | 0 | 1 | 0.22 |  | 0 |  | 0 |  | 0 |
| Soil/detritis |  |  |  | 1 | 0.01 |  | 0 |  | 0 |  | 0 |  | 0 | 1 | 0.09 |  | 0 | 1 | 0 |  | 0 |  | 0 |
| Rubbish |  | Paper, plastic, fishing line |  | 1 | 0.03 | 1 | 0.2 |  | 0 |  | 0 |  | 0 |  | 0 | 1 | 0.22 | 1 | 0.16 |  | 0 | 1 | 1.52 |

S3 Table legend.

**Data are presence records (‘1’ indicates present) based on all (raw and summary) data sources^1,2,3,4,5,6,7,8,9,10,11,12,13,14,16,18,19,20,21,22,23,24,25,26,27,28,29,30,3132,33,34,35,36,37,38,39,40,41,42,43,44,45,46,47,48,49,50,51,a,b,c,d,e,f,g,h,i,j,k,l,m,n,o,p,q, r,s,t,u,v,w,x,y,z,aa,bb,cc,dd,ee,ff,gg,hh,ii,jj,kk,ll,mm,nn,oo,pp,qq,rr,ss,tt,uu,vv,ww^, and percentage frequency occurrence (%) based on raw data sources^16,43,a,b,c,d,e,f,g,h,i,j,k,l,m,n,o,p,q,r,s,t,u,v,w,x,y,z,aa,bb,cc,dd,ee,ff,gg,hh,ii,jj,kk,ll,mm,nn,oo,pp,qq,rr,ss,tt,uu,vv,ww^ across all regions of Victoria (raw data: *n* = 11,569 scats; summary data^^: n = 11,103^^,¥,φ^ scats, *n* = 2523 stomachs, *n* = 67 scat/stomach) and within each region: Mallee (raw data: *n* = 5592 scats); Wimmera (raw data: *n* = 189 scats); Northern Country (raw data: *n* = 98 scats; summary data: *n* = 79 stomachs); North East (raw data: *n* = 2931 scats; summary data: *n* = 2501^¥^ scats, *n* = 14 stomachs, *n* = 67 scat/stomach); East Gippsland (G) (raw data: *n* = 1062 scats; summary data: *n* = 1624 scats^, *n* = 153 stomachs); West and South Gippsland (raw data: *n* = 461 scats; summary data: *n* = 26 scats, *n* = 92 stomachs); Central (raw data: *n* = 629 scats; summary data: *n* = 6747 scats; *n* = 391 stomachs); North Central (raw data: *n* = 181 scats; summary data^^); and South West (raw data: *n* = 308 scats; summary data: *n* = 144^φ^ scats, *n* = 197 stomachs).

^excluding Peacock et al. 1992 for which only total sample size for dog and fox of 110 provided

^^no sample size provided

^¥^den litter was also used to examine diet by Robinson et al. 1992

^φ^excludes Seebeck 1978 for which sample size was not provided

^ǂ^probable identification

*exotic species

^ƹ^species that have not previously been identified in the diet of foxes in Victoria in published studies that have used scat or stomach analysis

^€^Threatened (FFG Act 1988)

^£^Endangered (EPBC Act 1999)

^≠^Vulnerable (EPBC Act 1999)

^∂^Critically Endangered (IUCN 2013)

^∏^Endangered (IUCN 2013)

^∫^Near Threatened (IUCN 2013)

^ↄ^Vulnerable (IUCN 2013)

^Ω^Least Concern (IUCN 2013)

**References**

1. Baker GD, Degabriele R (1987) The diet of the red fox (*Vulpes vulpes*) in the Eldorado Hills of north-east Victoria Victorian Naturalist 104: 39-42.

2. Bertuch ID (1975) A mammal survey of Sherbrooke Forest Park using predator scat analysis: Latrobe University.

3. Brown GW, Triggs BE (1990) Diets of wild canids and foxes in East Gippsland 1983-1987, using predator scat analysis. Australian Mammalogy 13: 209-213.

4. Brown GW, Carr GW, Cherry KA, Craig SA, Horrocks GFB, et al. (1986) Flora and fauna of the Quadra forest block, East Gippsland, Victoria. Unpublished report. Melbourne: Department of Conservation, Forests and Lands.

5. Brown GW, Earl GE, Griffiths RC, Horrocks GFB, Williams LM (1989) Flora and fauna of the Acheron Forest Block, Central Highlands, Victoria. Unpublished report. Melbourne: Department of Conservation, Forests and Lands.

6. Brown GW, Horrocks GFB, Lunt ID, Meggs RA, Sandiford EM (1987) Flora and fauna of the Noorinbee forest block, East Gippsland, Victoria. Unpublished report. Melbourne: Department of Conservation, Forests and Lands.

7. Brown GW, Horrocks GFB, Meggs RA, Opie AM, Westaway J (1988) Flora and fauna of proposed timber harvesting areas in the Grampians National Park, Victoria. Part II. Unpublished report. Melbourne: Department of Conservation, Forests and Lands.

8. Brunner H (1978) The diet of dingoes and foxes in the Dartmouth Reservoir area. Victorian State Rivers and Water Supply Commission: Dartmouth Dam Project Report on Environmental Studies Part 2 pp. 19-28

9. Brunner H, Lloyd JW, Coman BJ (1975) Fox scat analysis in a forest park in south-eastern Australia. Australian Wildlife Research 2: 147–154.

10. Chesterfield EA, Hurley VA, Henry SR, Schulz M, Pyrke AF (1988) Flora and fauna of the Brodribb Forest block, East Gippsland, Victoria. Unpublished report. Melbourne: Department of Conservation, Forests and Lands.

11. Chesterfield EA, Macfarlane MA, Allen D, Hutchinson MN, Triggs B, et al. (1983) Flora and fauna of the Rodger Forest Block, East Gippsland, Victoria. Unpublished report. Forests Commission of Victoria.

12. Coates TD, Wright CJ (2003) Predation of the southern brown bandicoots *Isoodon obesulus* by the european red fox *Vulpes vulpes* in south-east Victoria. Australian Mammalogy 25: 107-110.

13. Coman BJ (1973) The diet of red foxes, *Vulpes vulpes* L., in Victoria. Australian Journal of Zoology 21: 391-401.

14. Corbett LK (1974) Contributions to the biology of dingoes (Camivora: Canidae) in Victoria. Melbourne: Monash University. 197 p.

15. Davies JB, Carter RL, Drummond MB, Hollis GJ, Pascoe CG, et al. (1994) Flora and fauna of the Eastern and Western Tyers Forest Blocks and adjacent south-eastern slopes of Baw Baw National Park, central Gippsland, Victoria. Unpublished report. Department of Conservation and Natural Resources.

16. Earl GE, Brown GW, Cherry KA, Horrocks GFB, Vollebergh PJ (1989) Flora and fauna of the Ellery Forest Block (southern and eastern parts), East Gippsland, Victoria. Unpublished report. Melbourne: Department of Conservation, Forests and Lands.

17. Gillespie GR, Henry SR, Mueck SG, Scotts D, Westaway J (1990) Flora and fauna of the Pheasant Creek and Upper Buenba forest blocks, alpine area, Victoria. Ecological Survey Report No. 29, Department of Conservatoin and Environment, Victoria. Unpublished report.

18. Gillespie GR, Humphries R, Horrocks GFB, Lobert BO, McLaughlin J (1992) Flora and fauna of the Stony Peak and Genoa Forest Blocks, East Gippsland, Victoria. Unpublished report. Melbourne: Department of Conservation and Environment, Victoria.

19. Henry SR, Cherry KA, Hurley VA, Opie AM, Schulz M (1988) Flora and fauna of the Tennyson forest block, east Gippsland, Victoria. Unpublished report. Melbourne: Department of Conservation, Forests and Lands.

20. Hollis GJ, Carter RL, Davies JB, Davies MJ, Drummond MB, et al. (1995) Survey of vegetation and vertebrate fauna in the Cascade Forest Block and adjacent areas of Baw Baw National Park, Central Gippsland, Victoria. Unpublished report. Department of Conservation and Natural Resources.

21. Hollis GJ, Carter RL, Davies JB, Drummond MB, Oates AM, et al. (1995) Survey of vegetation and vertebrate fauna in the Ada Forest Block, Port Phillip Area, Victoria. Unpublished report. Department of Conservation and Natural Resources.

22. Horrocks GFB, Opie AM, Carr GW, Cherry KA, Brown GW, et al. (1984) Flora and fauna of the Ellery Forest Block, East Gippsland, Victoria. Unpublished report. Melbourne: Department of Conservation, Forests and Lands.

23. Kirkwood R, Dann P, Belvedere M (2000) Effects of the seasonal availability of short-tailed shearwaters (*Puffinus tenuirostris*) on the diet of red foxes (*Vulpes vulpes*) on Phillip Island, Victoria. Australian Mammalogy 22: 87-92.

24. Kirkwood R, Dann P, Belvedere M (2005) A comparison of the diets of feral cats *Felis catus* and red foxes *Vulpes vulpes* on Phillip Island, Victoria. Australian Mammalogy 27: 89-93.

25. Lobert BO, Gillespie GR, Lunt ID, Peacock RJ, Robinson D (1991) Flora and fauna of the Goolengook Forest Block, East Gippsland, Victoria. Unpublished report. Melbourne: Department of Conservation and Environment, Victoria.

26. Loyn RH, Cameron DG, Traill BJ, Sloan JF, Malone BS, et al. (1992) Flora and fauna of the Cooaggalah Forest Block, East Gippsland, Victoria. Unpublished report. Melbourne: Department of Conservation and Environment, Victoria.

27. MacFarlane MA, Loyn RH, Chesterfield EA, Traill BJ, Triggs BE (1984) Flora and fauna of the Scorpion and Dawson Forest Blocks, East Gippsland, Victoria. Unpublished report. Melbourne: Department of Conservation, Forests and Lands.

28. Macfarlane MA, Schulz M, Parkes DM, Traill BJ, Triggs BE (1987) Flora and fauna of the Buckland forest block, east Gippsland, Victoria. Unpublished report. Melbourne: Department of Conservation, Forests and Lands.

29. Murrihy M (1991) A mammal survey of The Briars and Mt. Martha Park. Melbourne: Deakin University.

30. Norman FI (1971) Predation by the fox (*Vulpes vulpes* L.) on colonies of the short-tailed shearwater (*Puffinus tenuirostris* (Temminck)) in Victoria, Australia. Journal of Applied Ecology 8: 21-32.

31. Opie AM, Brown GW, Carr GW, Cherry KA, Horrocks GFB, et al. (1987) Flora and fauna of the Splitters Range forest block, Bairnsdale Region, Victoria. Unpublished report. Department of Conservation, Forests and Lands.

32. Opie AM, Cherry KA, Horrocks GFB, Carr GW, Schulz M, et al. (1984) Flora and fauna of the Yalmy forest block, East Gippsland, Victoria. Unpublished report. Melbourne: Department of Conservation, Forests and Lands.

33. Opie AM, Gillespie GR, Henry SR, Lobert BO, Pyrke AF (1990) Flora and fauna of the Coast Range Forest Block (southern part) East Gippsland, Victoria. Unpublished report. Melbourne: Department of Conservation and Environment.

34. Parkes DM, Macfarlane MA, Schulz M, Traill BJ (1987) Flora and fauna of the Nunniong North forest block, Bairnsdale region, Victoria. Unpublished report. Melbourne: Department of Conservation, Forests and Lands.

35. Peacock RJ, Brown GW, Duncan S, Gillespie GR, Robinson D, et al. (1992) Flora and fauna of the Sardine Forest Block, East Gippsland, Victoria. Unpublished report. Melbourne: Department of Conservation and Environment, Victoria.

36. Pyrke AF, Gillespie GR, Henry SR, Meggs RA, Westaway J (1988) Flora and fauna of the Clover and Pretty Valley Forest Blocks, north-east Victoria. Unpublished report. Melbourne: Department of Conservation, Forests and Lands.

37. Robinson PD, Collins MG, Gillespie GR, Humphries R, Lobert BO, et al. (1992) Flora and fauna of the Saltpetre Forest Block, north-east Victoria. Unpublished report. Melbourne: Department of Conservation and Environment, Victoria.

38. Robley A, Gormley A, Triggs B, Albert R, Bowd M, et al. (2014) Glenelg Ark 2005–2013: Evidence of the Benefits for Native Mammals of Sustained Fox Control. Arthur Rylah Institute for Environmental Research Technical Report Series. Department of Environment and Primary Industries, Heidelberg, Victoria.

39. Schulz M, Macfarlane MA, Parkes DM, Traill BJ, Triggs B, et al. (1987) Flora and fauna of the Mt Murray forest block, north-eastern Victoria. Unpublished report. Melbourne: Department of Conservation, Forests and Lands: Public Lands and Forest Division.

40. Seebeck JH (1978) Diet of the fox *Vulpes vulpes* in a western Victorian forest. Australian Journal of Ecology 3: 105-108.

41. Suckling GC, Backen E, Heislers A, Neuman FG (1976) The flora and fauna of radiata pine plantations in north-eastern Victoria. Forests Commission Victoria Bulletin 24: 1-58.

42. Triggs B, Brunner H, Cullen JM (1984) The food of fox, dog and cat in Croajingalong National Park, south-eastem Victoria. Australian Wildlife Research 11: 491-499.

43. Victoria College (1988) Forest regeneration, significant vegetation and small mammals in the Coast Range Forest Block, east Gippsland, Victoria. Unpublished reports. Faculty of Science, Victoria College, Melbourne.

44. Wallis RL, Brunner H (1986) Changes in mammalian prey of foxes, *Vulpes vulpes* (Carnivora:Vanidae) over 12 years in a forest park near Melbourne, Victoria. Australian Mammalogy 10: 434.

45. Wallis RL, Brown PR, Brunner H, Andrasek AM (1990) The vertebrate fauna of Dandenong Valley Metropolitan Park. A report prepared for the Melbourne and Metropolitan Board of Works, Waterways and Parks Division by Department of Science and Centre for Australian Applied Ecological Research, Victoria College, Melbourne.

46. Wallis RL, Brunner H, Seebeck JH (1996) The diet of red foxes and cats: their impact on fauna living in parks near Melbourne. Victorian Naturalist 113: 300-305.

47. Westaway J, Cherry KA, Duncan PE, Gillespie GR, Henry SR, et al. (1990) Flora and fauna of the Lower Wilkinson and Fainting Range forest blocks, Bairnsdale region, Victoria. Ecological Survey Report No. 27, Department of Conservation and Environment, Victoria. Unpublished report.

48. Westaway J, Henry SR, Gillespie GR, Lobert BO, Scotts DJ, et al. (1990) Flora and fauna of the west Errinundra and Delegate forest blocks, East Gippsland, Victoria. Unpublished report. Department of Conservation and Environment, Victoria.

49. White JG, Gubiani R, Smallman N, Snell K, Morton A (2006) Home range, habitat selection and diet of foxes (*Vulpes vulpes*) in a semi-urban riparian environment. Wildlife Research 33: 175–180.

50. Wilson BA, Wolrige J (2000) Assessment of the diet of the fox, *Vulpes vulpes*, in habitats of the eastern Otway Ranges, Victoria. Australian Mammalogy 21: 201-211.

51. Yugovic JV, Brown GW, Henry SR, Meggs RA (1987) Flora and fauna of the Sisters Forest Block, east Gippsland, Victoria. Unpublished report. Melbourne: Department of Conservation, Forests and Lands.

**Other data sources**

^a^Arthur Rylah Institue for Environmental Research, Department of Environment and Primary Industries (DEPI)

^b^Biosis

^c^DEPI

^d^DEPI Alexandra

^e^DEPI Bairnsdale

^f^DEPI Benalla

^g^DEPI Corryong

^h^DEPI Dartmoor

^i^DEPI Hamilton

^j^DEPI Horsham

^k^DEPI Mallacoota

^l^DEPI Mildura

^m^DEPI Orbost

^n^DEPI Portland

^o^DEPI Tallangatta

^p^DEPI Wangaratta

^q^DEPI Warragul

^r^DEPI Wodonga

^s^DEPI Yarram

^t^Ecology Australia

^u^Gippsland High Country Tours

^v^Melbourne Water

^w^Mornington Peninsula Shire

^x^Mt Buller & Mt Stirling Alpine Resort Management Board

^y^Parks Victoria (PV)

^z^PV Bairnsdale

^aa^PV Braeside

^bb^PV Cann River

^cc^PV Dargo

^dd^PV Dimboola

^ee^PV Foster

^ff^PV Gembrook

^gg^PV Halls Gap

^hh^PV Heyfield

^ii^PV Lysterfield-Berwick

^jj^PV Mallacoota

^kk^PV Mansfield

^ll^PV Mildura

^mm^PV Mt Buffalo

^nn^PV Sale

^oo^PV Tallangatta

^pp^PV Templestowe

^qq^PV Wangaratta

^rr^PV Wilsons Promontory National Park

^ss^Phillip Island Nature Park

^tt^Victoria Malleefowl Recovery Group

^uu^Wild Ecology

^vv^Wildlife Unlimited

^ww^Winton Wetlands Committee of Management
